# Near-IR absorbing donor–acceptor ligand-to-ligand charge-transfer complexes of nickel(ii)†
†Electronic supplementary information (ESI) available: Complete experimental procedures, 1H, 13C and VT NMR data, complete electrochemical and solvatochromic analysis, and frontier orbital energies and atomic positions from DFT calculations. CCDC 1414822–1414824. For ESI and crystallographic data in CIF or other electronic format see DOI: 10.1039/c5sc02703a



**DOI:** 10.1039/c5sc02703a

**Published:** 2015-12-08

**Authors:** Lindsay A. Cameron, Joseph W. Ziller, Alan F. Heyduk

**Affiliations:** a Department of Chemistry , University of California , Irvine , California 92697 , USA . Email: aheyduk@uci.edu

## Abstract

A new series of square-planar nickel(ii) donor–acceptor complexes exhibiting ligand-to-ligand charge-transfer (LL'CT) transitions have been prepared. Whereas the use of a catecholate donor ligand in conjunction with a bipyridyl acceptor ligand affords a complex that absorbs throughout the visible region, the use of a azanidophenolate donor ligands in conjunction with a bipyridyl acceptor ligand affords complexes that absorbs well into the near-IR region of the solar spectrum. Three new complexes, (cat)Ni(bpy^*t*^Bu_2_) (**1**; (cat)^2–^ = 3,5-di-*tert*-butyl-1,2-catecholate; bpy^*t*^Bu_2_ = 4,4′-di-*tert*-butyl-2,2′-bipyridine), (ap)Ni(bpy^*t*^Bu_2_) (**2**; (ap)^2–^ = 4,6-di-*tert*-butyl-2-(2,6-diisopropylphenylazanido)phenolate), and (ap^Ph^)Ni(bpy^*t*^Bu_2_) (**3**; (ap^Ph^)^2–^ = 10-(2,6-diisopropylphenylazanido)-9-phenanthrolate), have been prepared and characterized by structural, electrochemical, and spectroscopic methods. Whereas all three square-planar complexes show multiple reversible one-electron redox-processes and strong LL'CT absorption bands, in azanidophenolate complexes **2** and **3**, the LL'CT absorption covers the near-IR region from 700–1200 nm. The electronic absorption spectra and ground state electrochemical data for **2** and **3** provide an estimate of their excited-state reduction potentials, *E* ^+/^*, of –1.3 V *vs.* SCE, making them as potent as the singlet excited state of [Ru(bpy)_3_]^2+^.

## Abstract 2

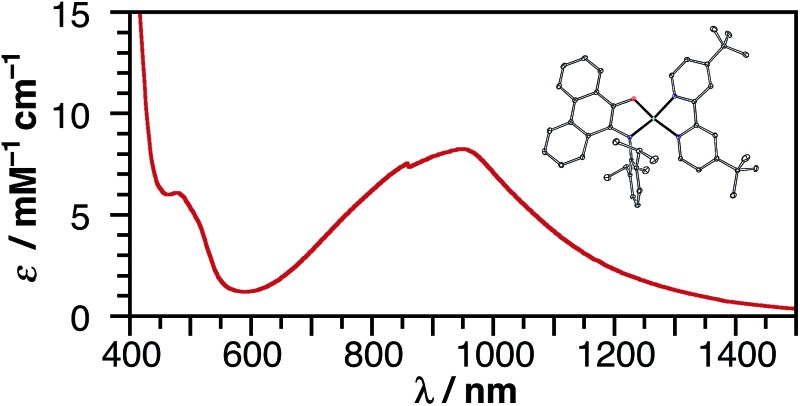
Ligand-to-ligand charge-transfer complexes of nickel(ii) incorporating azanidophenolate donor ligands absorb near-IR light to access highly reducing excited states.

## Introduction

Ligand-to-ligand charge-transfer (LLCT) complexes are a class of charge-transfer complex in which the frontier photoactive molecular orbitals are localized primarily on the ligand set, with the metal valence orbitals displaced to lower or to higher energy.^1–10^ Commonly, LLCT complexes of transition metal ions incorporate redox-active ligands, since these ligands have HOMO and LUMO energies that insert in between the filled and empty metal d-orbitals.^11^ The diversity of redox-active ligands available, and the ability to readily modify existing ligands with functional groups to change their electronic and steric characteristics, allows the properties of LLCT complexes to be tuned over a broad range.^12,13^ In this way, the absorption profile, redox potentials, and molecular polarity of LLCT complexes can be readily modified so the complexes can be used for diverse applications such as photochemical charge-transfer and non-linear optics.^14–21^

The ideal structural motif for LLCT complexes is the four-coordinate, square-planar complex.^8^ This coordination environment allows the redox-active ligands to be coplanar, thus maximizing the intensity of the optical LLCT transition that moves electrons between two ligands. Square-planar LLCT complexes come in two limiting extremes: symmetrical LLCT complexes that have two of the same (or very similar) redox-active ligands coordinated to the metal ion, and unsymmetrical or donor–acceptor LL'CT^22^ complexes that have two different redox-active ligands coordinated to the metal ion.

An example of the symmetrical class of LLCT complexes are the *ortho*-iminosemiquinonate complexes of the Group 10 metals nickel, palladium, and platinum, M^II^(isq˙)_2_.^23–31^ In these complexes, the redox-active ligands are identical, and adopt an open-shell radical monoanionic oxidation state. The M^II^(isq˙)_2_ complexes are characterized by a very intense LLCT transition that appears in the visible to near-IR portion of the electromagnetic spectrum. These symmetrical LLCT complexes are non-polar both in the ground state and in the excited state and as such the symmetric LLCT transition has significant π to π* character.^32–34^

Donor–acceptor LL'CT complexes are exemplified by complexes such as (cat)Pt(bpy) (cat^2–^ = catecholate, bpy = 2,2′-bipyridine).^14,35–37^ In this case, the catecholate is a dianion and acts as the donor ligand while the bipyridine is neutral and acts as the acceptor ligand. Donor–acceptor LL'CT complexes are dipolar in the ground state and are characterized by a LL'CT band in the visible region that corresponds to the transfer of an electron from the donor ligand to the acceptor ligand affording a non-polar (or less polar) excited state. It is this latter category of LL'CT complexes that is of most interest, as the photo-induced change in polarity can give rise to interesting non-linear optical properties. Furthermore, the directional nature of the LL'CT transition in donor–acceptor complexes gives the molecule photodiode-like properties, making them attractive candidates as charge-transfer photosensitizers.^20,21^ Square-planar donor acceptor complexes of platinum are well established in the literature. In particular, examples that combine catecholate, dithiolate, or acetylide donor ligands with bipyridine or phenanthroline acceptor ligands are well known.^5,6,9,14,35–45^ Strong metal-ligand π interactions in platinum complexes of the softer dithiolate and acetylide ligands results in significant mixing of the platinum d-orbitals and ligand π-symmetry orbitals.^42–45^ This mixing results in the optical charge-transfer transition taking on significant metal character: that is a mixed-metal-ligand-to-ligand charge-transfer (MMLL'CT) transition.^9,43–45^ The dithiolate and acetylide derivatives are emissive in solution owing to a large spin–orbit coupling contribution from platinum that facilitates intersystem crossing to a long-lived ^3^MMLL'CT excited state. The long-lived ^3^MMLL'CT excited state of these complexes allows them to be used as photosensitizers either in homogeneous solution (bimolecular electron or energy transfer) or covalently linked to an electron collector (intramolecular electron transfer).^46,47^

**Chart 1:**
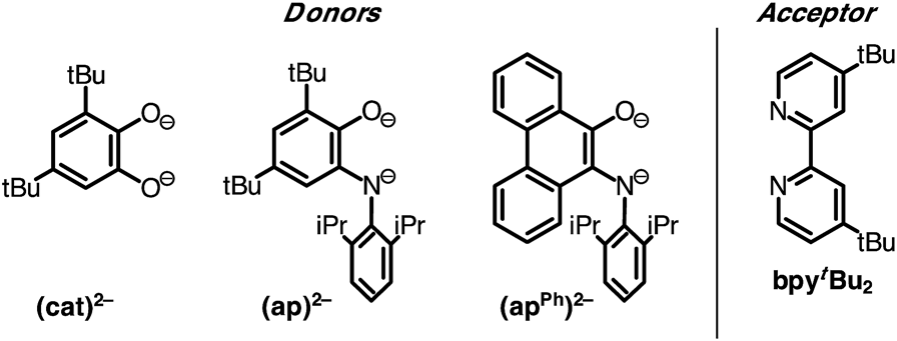
Donor and acceptor ligands used in this study.

Despite the preponderance of donor–acceptor LL'CT complexes of platinum, much less is known about the analogous complexes of nickel.^5,12,48–51^ The development of such complexes would be of interest, especially in the development of earth-abundant photosensitizers for application to dye-sensitized solar cells (DSSCs). Herein we report the synthesis and characterization of a new class of donor–acceptor LL'CT complexes of nickel(ii) that incorporate catecholate or highly-reducing azanidophenolate^52^ donor ligands with bipyridine-type acceptor ligands (Chart 1). Incorporation of the azanidophenolate donor pushes the LL'CT absorption band well into the near-IR portion of the spectrum, a region that is challenging for traditional charge-transfer chromophores. Single-crystal X-ray diffraction studies were used to establish the structural properties of the donor–acceptor molecules while steady-state electronic spectroscopy, electrochemistry, and DFT computations were used to establish their electronic properties. Negative solvatochromism^9^ confirms the donor–acceptor nature of the complexes even when the LL'CT transition is pushed out to the near-IR region of the spectrum. Spectroscopic and electrochemical data show that the LL'CT excited states in these “red” dyes are still strongly reducing, suggesting that these complexes are attractive candidates for charge-transfer photosensitizers in panchromatic solar cells.

## Results and discussion

### Synthesis and structural characterization

**Scheme 1:**
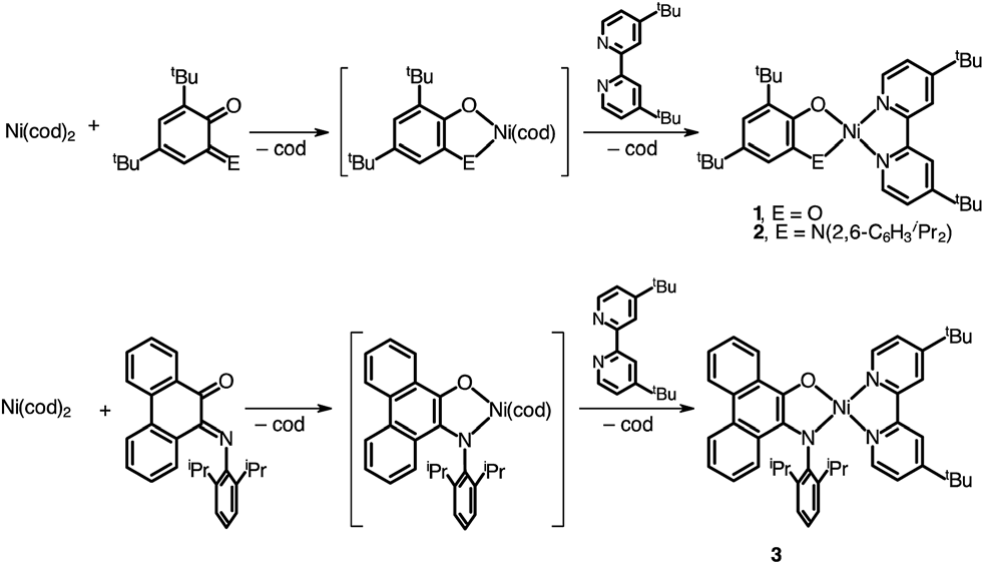


A family of square-planar nickel(ii) charge-transfer complexes has been prepared by exploiting the two-electron oxidative properties of *ortho*-quinones and *ortho*-iminoquinones. Previously, we reported the synthesis of square-planar nickel(ii) complexes that were accessed by the reaction of the nickel(0) synthon, Ni(cod)_2_ (cod = 1,4-cyclooctadiene), with *ortho*-quinones.^12^ Following this strategy, Ni(cod)_2_ was combined with one equivalent of 3,5-di-*tert*-butyl-1,2-quinone, and the mixture was then treated with one equivalent of 4,4′-di-*tert*-butyl-2,2′-bipyridine (bpy^*t*^Bu_2_) (Scheme 1). A dark blue solution formed immediately, which produced the square planar nickel complex, (cat)Ni(bpy^*t*^Bu_2_) (**1**; (cat)^2–^ = 3,5-di-*tert*-butyl-1,2-catecholate) in 80% yield. While *ortho*-quinones are relatively potent oxidants, the replacement of one quinone oxygen atom with a nitrene in an *ortho*-iminoquinone is known to significantly reduce this oxidizing ability.^52^ Nevertheless, initial treatment of Ni(cod)_2_ with *ortho*-iminoquinone followed by the addition of bpy^*t*^Bu_2_, led to the formation of highly-colored products characteristic of square-planar (donor)Ni(acceptor) complexes. In this way, the new complexes (ap)Ni(bpy^*t*^Bu_2_) (**2**; (ap)^2–^ = 4,6-di-*tert*-butyl-2-(2,6-diisopropylphenylazanido)phenolate) and (ap^ph^)Ni(bpy^*t*^Bu_2_) (**3**; (ap^ph^)^2–^ = 10-(2,6-diisopropylphenylazanido)-9-phenanthrolate) were prepared as brightly-colored microcrystalline solids in 66% and 81% yields, respectively.

**Fig. 1:**
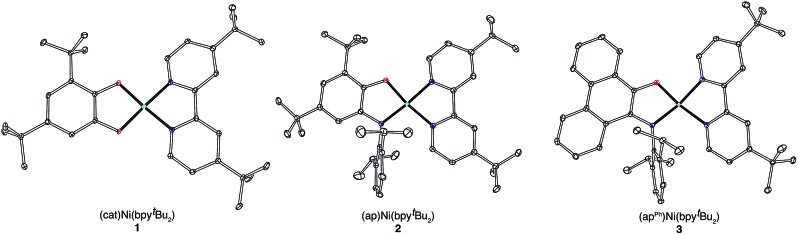
ORTEP diagrams of (cat)Ni(bpy^*t*^Bu_2_) (**1**), (ap)Ni(bpy^*t*^Bu_2_) (**2**), and (ap^Ph^)Ni(bpy^*t*^Bu_2_) (**3**). Thermal ellipsoids are shown at 50% probability. Hydrogen atoms and non-coordinated solvent molecules have been omitted for clarity.

High-resolution single-crystal X-ray diffraction studies were used to confirm gross structural features and to elucidate the finer points of oxidation state and electron distribution in all three new nickel complexes. Fig. 1 shows the ORTEP diagrams of (cat)Ni(bpy^*t*^Bu_2_) (**1**), (ap)Ni(bpy^*t*^Bu_2_) (**2**), and (ap^ph^)Ni(bpy^*t*^Bu_2_) (**3**); Table 1 lists selected bond distances within the primary coordination sphere of each complex. All three complexes display square-planar geometry, consistent with diamagnetic, *d* ^8^ nickel(ii) metal centers. The bite angles of both chelating ligands are consistent throughout the series with the catecholates adopting an O–Ni–O bite angle of 89° and the azanidophenolates adopting an O–Ni–N bite angle of 86°. Across the three complexes the bpy^*t*^Bu_2_ ligands display N–Ni–N bite angles in the 82–83° range. The bpy^*t*^Bu_2_ ligands of all three complexes show bridgehead C(7)–C(8) bond distances of 1.46–1.47 Å and C–N distances of 1.35–1.37 Å, consistent with neutral bipyridyl-type ligands.^53^ The donor ligands in **1**, **2**, and **3** all show C–O distances of 1.34–1.36 Å, whereas for the azanidophenolate donor ligands of **2** and **3** show C–N distances of 1.39 Å. These values for both the catecholate and azanidophenolate ligands are consistent with doubly-reduced, dianionic form of the ligands, which is further supported by ligand metrical oxidation states (MOS) of –1.90 and –1.89 for **1** and **2**, respectively.^54–56^ Thus, the available structural data strongly supports a (donor)Ni^II^(acceptor) electronic structure for **1**, **2**, and **3**, with either a dianionic catecholate or a dianionic azanidophenolate ligand acting as the electron-rich donor and a neutral bpy^*t*^Bu_2_ ligand acting as the electron-poor acceptor.

**Table 1:** Selected bond distances (Å) for (cat)Ni(bpy^*t*^Bu_2_) (**1**), (ap)Ni(bpy^*t*^Bu_2_) (**2**), and (ap^Ph^)Ni(bpy^*t*^Bu_2_) (**3**), including the calculated metrical oxidation state (MOS)

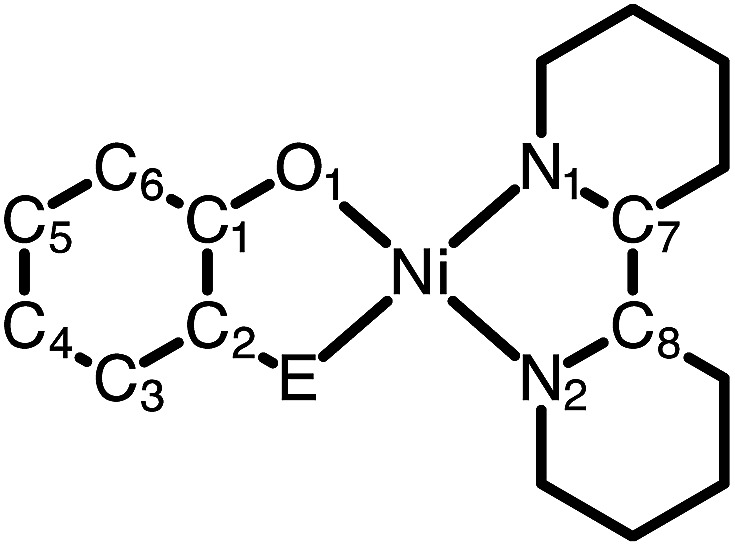			
Bond distances/Å			
	**1**	**2**	**3**
Ni–O_1_	1.8227(11)	1.8329(17)	1.8212(8)
Ni–E	1.8273(11)^*a*^	1.868(2)^*b*^	1.8798(10)^*b*^
Ni–N_1_	1.8804(14)	1.908(2)	1.9048(10)
Ni–N_2_	1.8868(13)	1.942(2)	1.9350(10)
O_1_–C_1_	1.3574(18)	1.360(3)	1.3401(14)
E–C_2_	1.3538(18)^*a*^	1.389(3)^*b*^	1.3936(14)^*b*^
C_1_–C_2_	1.407(2)	1.404(3)	1.3871(16)
C_2_–C_3_	1.390(2)	1.398(3)	1.4537(16)
C_3_–C_4_	1.400(2)	1.387(4)	1.4374(16)
C_4_–C_5_	1.400(2)	1.390(4)	1.4512(17)
C_5_–C_6_	1.405(2)	1.411(3)	1.4222(17)
C_1_–C_6_	1.408(2)	1.392(3)	1.4224(16)
N_1_–C_8_	1.3569(19)	1.346(3)	1.3496(15)
N_2_–C_9_	1.3614(19)	1.358(3)	1.3659(15)
C_7_–C_8_	1.471(2)	1.459(3)	1.4692(16)
MOS^*c*^	–1.90	–1.89	N/A

^*a*^E = O.

^*b*^E = N of N(2,6-C_6_H_3_^i^Pr_2_).

^*c*^See ref. 56.

Consistent with the single-crystal X-ray data, catecholate complex **1** shows diamagnetic ^1^H and ^13^C NMR spectra indicating that the *d* ^8^ square-planar nickel geometry observed in the solid-state structure is preserved in solution. Whereas the unsymmetrical nature of the 3,5-di-*tert*-butyl-1,2-catecholate ligand should impart *C*_s_ symmetry on complex **1**, only a single sharp resonance is observed for the *tert*-butyl proton resonances of the bpy^*t*^Bu_2_ ligand at room temperature. We were unable to resolve these *tert*-butyl resonances using either a higher field NMR spectrometer or lower acquisition temperatures; however, the methyl and the tertiary carbon regions of the ^13^C NMR spectrum of **1** showed clear evidence for four distinct *tert*-butyl groups, consistent with the expected *C*_s_ symmetry of the square-planar complex.

The ^1^H NMR spectra of azanidophenolate complexes **2** and **3** are consistent with the expected *C*_s_ symmetry of these complexes, but show dynamic behavior that was resolved with variable-temperature ^1^H NMR studies. The isopropyl groups of the azanidophenolate ligands manifest an apparent septet near 4.5 ppm for the methine proton and a pair of doublets near 1.2 and 1.3 ppm for the chemically different methyl protons. The resonances for the aromatic protons of the azanidophenolate ligands appear upfield of the resonances for the aromatic protons of the bpy^*t*^Bu_2_ ligand, consistent with the more electron-rich nature of the azanidophenolate donor. At room temperature, the ^1^H NMR resonances for the aromatic protons of both the azanidophenolate and the bpy^*t*^Bu_2_ ligands of complexes **2** and **3** appear sharp and well resolved, consistent with the *C*_s_ symmetry, square-planar geometry indicated in the single-crystal X-ray structure. In contrast, at room temperature, the *tert*-butyl resonances of the bpy^*t*^Bu_2_ ligand of both **2** and **3** suggested fluxional behavior in solution. The ^1^H NMR resonances for the *tert*-butyl groups in the bpy^*t*^Bu_2_ ligand of **2** appear as two broad singlets at 0.72 and 0.91 ppm. In the case of **3**, these same ^1^H NMR resonances appear as a broad singlet at 0.86 ppm. The broadness of these resonances, and the fact that only one resonance is observed for **3**, are indicative of fluxional behavior in solution, prompting a variable-temperature (VT) ^1^H NMR spectroscopic study.

**Fig. 2:**
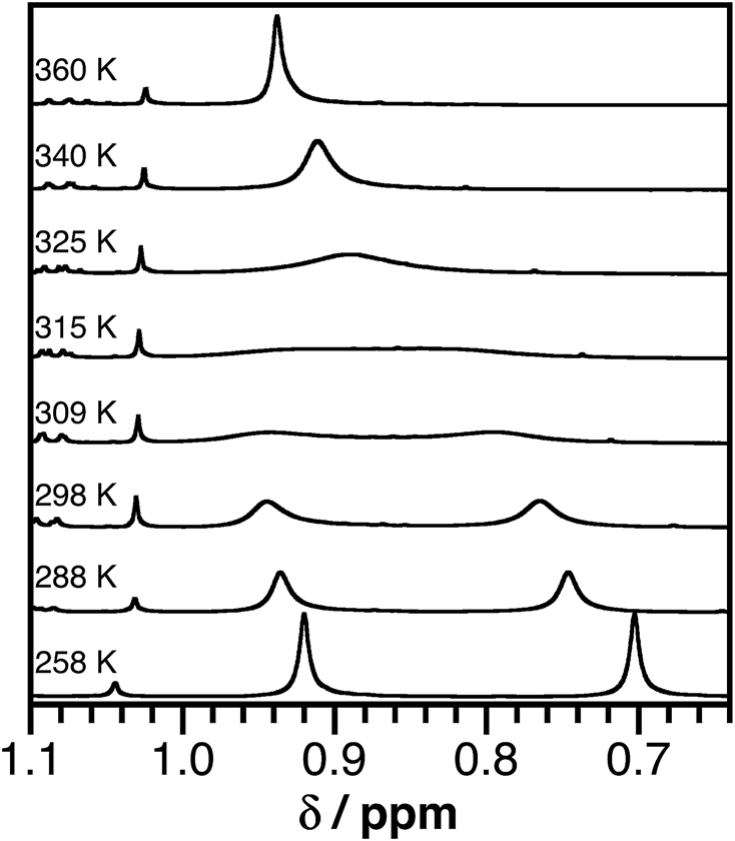
Partial ^1^H NMR spectra (500 MHz) of (ap)Ni(bpy^*t*^Bu_2_) (**2**) in toluene-*d*_8_ showing the *tert*-butyl proton resonances of the bpy^*t*^Bu_2_ ligand over the temperature range 258–360 K.

Fig. 2 shows ^1^H NMR spectra of the *tert*-butyl groups of the (bpy^*t*^Bu_2_) ligand of **2** in toluene-*d*_8_ over the temperature range 258–360 K. The temperature dependence of the NMR resonance was modeled using the complete band shape method for a simple two-site degenerate exchange.^57^ A linear temperature correction for the chemical shift at each temperature was applied and a transverse relaxation time (*T*_2_) of 0.08 s was estimated from the peak-width at half maximum. First-order rate constants for the exchange were estimated between 288 K and 340 K and these values were used to construct an Eyring plot (see ESI†) that afforded transition state energies of Δ*H* ^‡^ = 19.1 ± 1.5 kcal mol^–1^ and Δ*S* ^‡^ = 12 ± 5 cal mol^–1^ K^–1^ for **2**. A similar analysis for **3** afforded Δ*H* ^‡^ = 17.6 ± 1.4 kcal mol^–1^ and Δ*S* ^‡^ = 15 ± 5 cal mol^–1^ K^–1^. While such activation parameters are often attributed to ligand dissociation processes, reactions between **2** and excess 2,2′-bipyridine indicate that complete dissociation of the acceptor ligand is not responsible for the dynamic behavior observed in the VT NMR experiments. Instead, the dynamic behavior is likely an intramolecular isomerization process. One possibility would be a molecular twist that affords a tetrahedral transition state. A second possibility would be for the dissociation of one arm of either the donor or the acceptor ligand to give a three-coordinate intermediate (without complete loss of the ligand). At elevated temperatures, isomerization by either process could be fast enough that the two sides of the bpy^*t*^Bu_2_ ligand are equivalent on the NMR timescale.

### Spectroscopy and electrochemistry

**Fig. 3:**
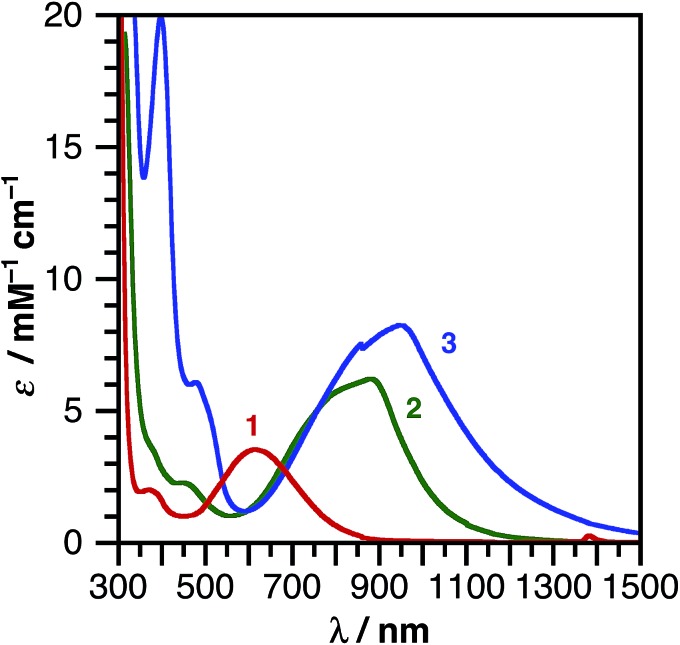
UV-vis-NIR absorption spectra of (cat)Ni(bpy^*t*^Bu_2_) (**1**), (ap)Ni(bpy^*t*^Bu_2_) (**2**), and (ap^Ph^)Ni(bpy^*t*^Bu_2_) (**3**) dissolved in THF at 298 K.

Catecholate complex **1** and azanidophenolate complexes **2** and **3** are highly colored both in the solid state and in solution, reflecting strong absorption bands in the UV-vis-NIR region of the electromagnetic spectrum. Fig. 3 shows the absorption spectra for these complexes dissolved in THF at 298 K. The band maxima (*λ*_max_), estimated excited-state energy (E_LL'CT_), extinction coefficient (*ε*), and solvatochromic shift of each complex are summarized in Table 2. A common feature among these complexes is the strong, low-energy absorption band that shifts from the visible portion of the spectrum with the catecholate donor ligand to the near-IR portion of the spectrum with the azanidophenolate donor ligands. Based on comparisons to the spectra of previously reported (catecholate)M(diimine) complexes (M = Ni, Cu),^12,58^ these absorptions are assigned as LL'CT transitions, owing to dominant contributions from the azanidophenolate and diimine ligands and minimal contributions from the nickel center (also see computations below). The LL'CT transitions show a strong negative solvatochromic response, appearing at higher energy in polar solvents and at lower energy in non-polar solvents, consistent with an excited state electronic structure that is less dipolar than the ground state electronic structure.^9^ In addition to the low-energy LL'CT transition, complex **3** with the phenanthrenediolate donor ligand shows intense absorption bands in the 300–600 nm region that are characteristic of phenanthrene π–π* transitions.^59^

**Table 2:** LL'CT absorption maxima, extinction coefficients and estimated excited state energies for (cat)Ni(bpy^*t*^Bu_2_) (**1**), (ap)Ni(bpy^*t*^Bu_2_) (**2**), and (ap^Ph^)Ni(bpy^*t*^Bu_2_) (**3**) in THF

	*λ*_max_/nm	*ε*/M^–1^ cm^–1^	*E*_LL'CT_/eV	Solvatochromic shift^*a*^
**1**	620	3600	1.52	0.45
**2**	890	6200	1.12	0.16
**3**	970	8100	0.95	0.19

^*a*^Determined using the electronic solvent number. See ESI and ref. 9.

**Fig. 4:**
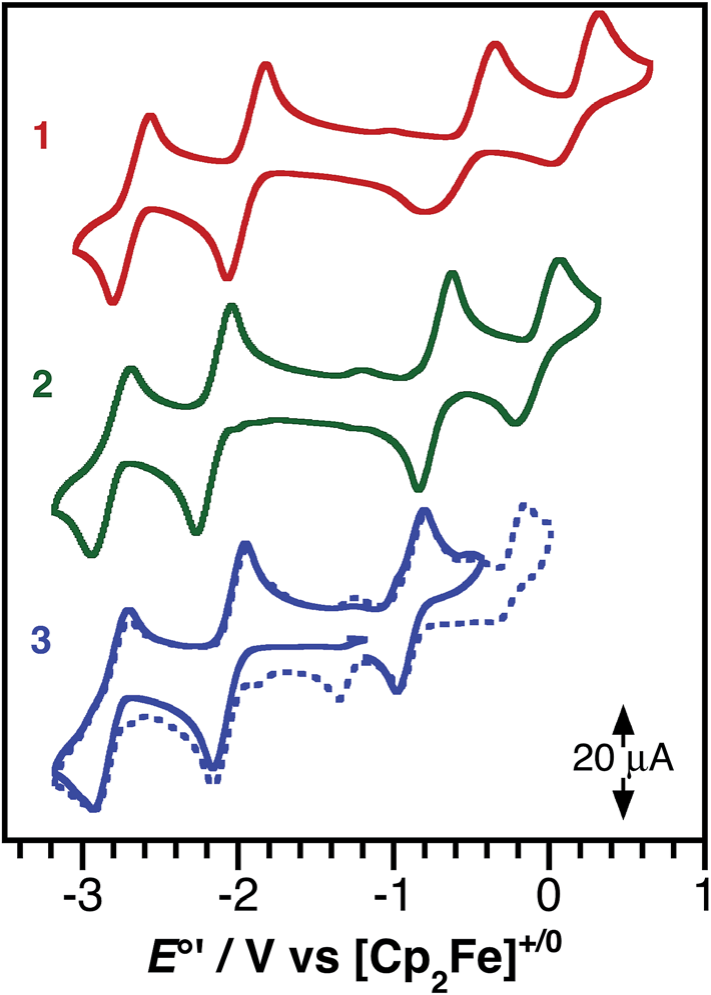
Cyclic voltammograms of (cat)Ni(bpy^*t*^Bu_2_) (**1**), (ap)Ni(bpy^*t*^Bu_2_) (**2**), and (ap^Ph^)Ni(bpy^*t*^Bu_2_) (**3**) as 1 mM solutions in THF containing 0.1 M [Bu_4_N][PF_6_] supporting electrolyte. Data were collected at a glassy carbon working electrode, with a platinum wire counter electrode, and a silver wire pseudo-reference electrode using a scan rate of 200 mV s^–1^.

The ground-state redox properties of the nickel charge-transfer complexes were probed by cyclic voltammetry. Fig. 4 shows the CVs of all three complexes and Table 3 summarizes the electrochemical potentials for two one-electron reductions and two one-electron oxidations for each nickel complex. All potentials were measured relative to the [Cp_2_Fe]^+/0^ couple using an internal standard. Notably, complexes **1–3** all contain the same bpy^*t*^Bu_2_ acceptor ligand and all show similar potentials for the first reduction process, *E*′_3_° [Ni]^0/–^, of –2.07 ± 0.07 V. In contrast, the first oxidation potential for **1–3**, *E*′_2_° [Ni]^+/0^, is highly sensitive to the identity of the donor ligand. Complex **1**, (cat)Ni(bpy^*t*^Bu_2_), with the catecholate donor ligand, is the most difficult to oxidize with an *E*′_2_° of –0.46 V *vs.* [Cp_2_Fe]^+/0^. This oxidation is only partially reversible, as the return wave is both broad and cathodically shifted. The irreversibility may stem from coordination of a solvent molecule to the putative cation, [(sq˙)Ni(bpy^*t*^Bu_2_)]^+^, during the course of the voltammetric sweep. Replacing one oxygen of the catecholate with a nitrene in **2**, resulted in an cathodic shift of *E*′_2_° by 270 mV to –0.73 V; whereas, the more conjugated phenanthroline backbone of **3** shifts *E*′_2_° even further to –0.90 V. Unlike **1**, the first oxidation of **2** and **3** shows good reversibility (*i*_pc_/*i*_pa_ = 1), possibly because the bulky 2,6-C_6_H_3_^i^Pr_2_ groups of the azanidophenolate ligands prevent solvent from coordinating to the metal center in the putative cation.

**Table 3:** Electrochemical data for (cat)Ni(bpy^*t*^Bu_2_) (**1**), (ap)Ni(bpy^*t*^Bu_2_) (**2**), and (ap^Ph^)Ni(bpy^*t*^Bu_2_) (**3**)

	*E*′°/V *vs.* [Cp_2_Fe]^+/0^				
	*E*′_1_° [Ni]^2+/1+^	*E*′_2_° [Ni]^+/0^	*E*′_3_° [Ni]^0/–^	*E*′_4_° [Ni]^1–/2–^	*E*′_2_°–*E*′_3_°
**1**	0.13	–0.46	–2.01	–2.76	1.54
**2**	–0.07	–0.73	–2.15	–2.80	1.42
**3**	–0.07	–0.90	–2.05	–2.80	1.15

The difference between the first oxidation and the first reduction, *E*′_2_°–*E*′_3_°, measures the thermodynamic HOMO–LUMO gap of the complexes. As shown in Table 3, the trend in *E*′_2_°–*E*′_3_° for complexes **1–3** follows with the changes to the donor ligand and furthermore, this trend mirrors the trend in the optical LL'CT band energy, observed in the electronic absorption spectrum.

### DFT computations

Density functional theory (DFT) computations were used to model the electronic structures of complexes **1–3**. The single-crystal structures were used as the starting point for geometry minimizations, which were refined initially at the TPSS/TZVP level of theory. All complexes refined as a closed-shell, *S* = 0, ground-state species. In general, the computed structures agreed well with the solid-state structures (see ESI†) with Ni–O, Ni–N, and intraligand bond distances within 0.02 Å of the solid-state data.

**Fig. 5:**
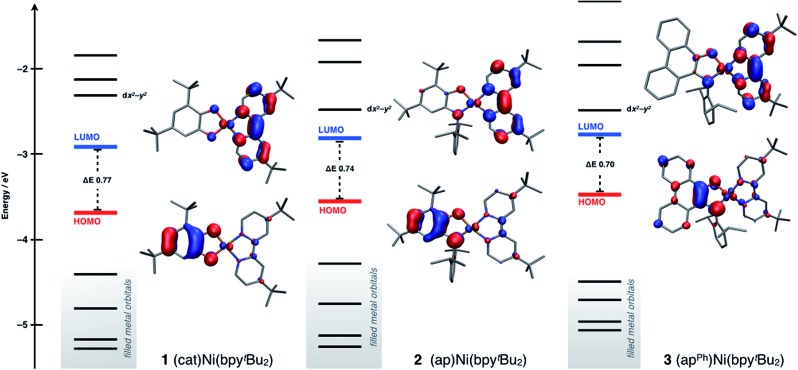
Frontier Kohn–Sham orbital diagram for (cat)Ni(bpy^*t*^Bu_2_) (**1**), (ap)Ni(bpy^*t*^Bu_2_) (**2**), and (ap^Ph^)Ni(bpy^*t*^Bu_2_) (**3**) as determined by DFT computations at the TPSS/TZVP level of theory.

The main goals of the computational studies were to evaluate the relative energies and to determine the electron distributions of the frontier orbitals for complexes **1–3** in the ground electronic state. Fig. 5 shows the frontier Kohn–Sham orbital diagram for complexes **1–3** along with depictions of the HOMO and LUMO for each complex. For all three complexes the HOMO is relatively high in energy and well isolated from other molecular orbitals, consistent with the electrochemical data that shows these complexes can be oxidized at modest potentials. The LUMO of complex **1** is also well isolated from higher-energy orbitals, but interestingly, for complexes **2** and **3** the LUMO+1 drops in energy, so that it is relatively close to the LUMO. The LUMO+1 is predominantly *d*_*x*^2^–*y*^2^_ and M–L σ* in character, so this result suggests that nickel centers in **2** and **3** experience a weaker ligand field than the nickel center in **1**. Consideration of the bond distances in Table 1 show that this result stems from steric rather than electronic considerations as all metal ligand bond distances are elongated in **2** and **3**. Regardless, across the series **1–3** the frontier orbitals remain localized on the ligands and not the nickel metal center. Consistent with both the spectroscopic and electrochemical data above, the calculated HOMO–LUMO gap decreases along the series **1** > **2** > **3**, though the magnitude of the decrease is smaller for the computation than for either measurement.

The HOMO and LUMO pictures of Fig. 5 support the assignment of complexes **1–3** as donor–acceptor molecules in which the frontier electronic structure is dominated by the ligand scaffold. Table 4 presents the percentage contribution from the donor ligand, from the bpy^*t*^Bu_2_ acceptor ligand, and from the nickel center to the HOMO and LUMO of each complex as determined by a Mulliken population analysis (MPA). In all three complexes, the HOMO and LUMO contain less than 11% metal character. The HOMO of each complex contains greater than 70% donor ligand character with catecholate complex **1** showing the greatest HOMO localization at 79.1% on the catecholate ligand. The LUMO of each complex has at least 74% bpy^*t*^Bu_2_ ligand character, again with complex **1** showing the highest degree of localization with almost 81% of the LUMO localized on the bpy^*t*^Bu_2_ ligand. Thus, according to the data in Table 4, complex **1** should be the most dipolar, consistent with the calculated dipole moment of 10.5 Debye. Complexes **2** and **3** are less dipolar, but still have calculated dipole moments of 8.9 Debye. These computational results are consistent with the solvatochromic shifts given in Table 2, which indicate that complex **1** has the most dipolar ground state. The localizations of the HOMO and LUMO, as well as the calculated dipole moments, are all consistent with the characterization of complexes **1–3** as donor–acceptor LL'CT complexes.

**Table 4:** Metal and ligand contributions to the HOMO and LUMO of (cat)Ni(bpy^*t*^Bu_2_) (**1**), (ap)Ni(bpy^*t*^Bu_2_) (**2**), and (ap^Ph^)Ni(bpy^*t*^Bu_2_) (**3**) as determined by Mulliken population analysis

	Orbital	Percentage contribution			Energy/eV
		Ni	Donor ligand	bpy^*t*^Bu_2_	
**1**	LUMO	8.1	10.9	80.9	–2.91
	HOMO	5.8	79.1	15.0	–3.68
**2**	LUMO	8.2	14.4	77.4	–2.82
	HOMO	7.2	74.2	18.6	–3.56
**3**	LUMO	10.4	15.3	74.3	–2.78
	HOMO	8.7	72.0	19.2	–3.48

## Conclusions

The development of charge-transfer complexes using earth-abundant metals is an important goal in solar-energy conversion strategies. In this regard, LL'CT complexes of earth-abundant metals are attractive due to the tunable nature of their electrochemical and photo physical properties, yet they remain surprisingly underdeveloped. By leveraging the tuneable nature of LL'CT transitions, new charge-transfer complexes have been developed with strong absorption into the near-IR portions of the solar spectrum that also generate strongly-reducing excited states. These charge-transfer complexes were constructed using a non-precious metal and readily accessible donor and acceptor ligands. Table 5 summarizes the estimated excited-state redox potentials for complexes **1–3** based on the absorption data of Table 2 and the electrochemical data of Table 3 (converted to the SCE scale by assuming a formal potential for [Cp_2_Fe]^+/0^ of +0.56 V *vs.* SCE).^60^

**Table 5:** Estimated excited-state redox potentials (V *vs*. SCE) for (cat)Ni(bpy^*t*^Bu_2_) (**1**), (ap)Ni(bpy^*t*^Bu_2_) (**2**), and (ap^Ph^)Ni(bpy^*t*^Bu_2_) (**3**)

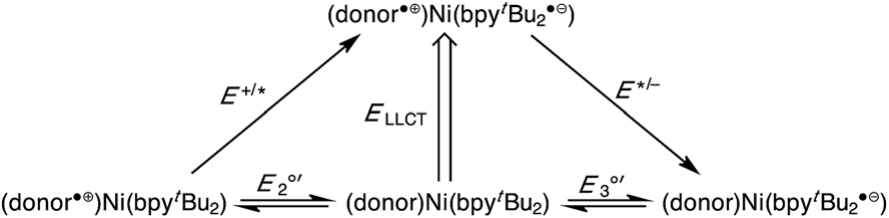		
(donor)Ni(bpy^*t*^Bu_2_)	*E* ^+/^*/V *vs.* SCE	E*^/–^/V *vs.* SCE
(cat)Ni(bpy^*t*^Bu_2_) (**1**)	–1.42	+0.07
(ap)Ni(bpy^*t*^Bu_2_) (**2**)	–1.29	–0.47
(ap^Ph^)Ni(bpy^*t*^Bu_2_) (**3**)	–1.29	–0.54

The potency of **1–3** as excited state reductants is revealed upon comparison to the ruthenium(ii) bipyridine family of MLCT complexes. The parent ruthenium dye, [Ru(bpy)_3_]^2+^, is a strong excited state reductant upon absorption of a visible photon (*λ*_max_ = 500 nm or 2.5 eV) with *E* ^+/^*(^1^MLCT) = –1.2 V *vs.* SCE.^60,61^ It has long been recognized that improved efficiencies in solar-energy conversion devices such as dye-sensitized cells could be possible with dyes that extend further into the near-IR;^62,63^ however, even the ruthenium “black dye” cuts off at 920 nm (1.35 eV).^64^ Catecholate complex **1** is a stronger excited-state reductant than [Ru(bpy)_3_]^2+^, and absorbs in the same spectral range (500–800 nm).^8^ Azanidophenolate complexes **2** and **3** access excited states that are just as reducing as [Ru(bpy)_3_]^2+^, but do so with light absorption profiles that cover the near-IR portions of the spectrum (700–1200 nm).

Across both the ruthenium MLCT family of dyes and the nickel LL'CT dyes presented here, the strongly reducing excited state derives from the incorporation of a bipyridyl-type acceptor ligand. Thus the “active” reducing electron in 1*, 2*, or 3* is held in the same type of bpy π* orbital that it is in the singlet excited state of [Ru(bpy)_3_]^2+^. The difference in **2** and **3** lies in their absorption profiles, since 2* and 3* are generated with much lower energy light. The red-shifted absorption profiles of **2** and **3** were possible because of the electron-rich nature of the azanidophenolate ligand, which results in a high-energy donor HOMO for the complex that lies well above the filled metal-based orbitals of the *d* ^8^ nickel(ii) center. It is worth noting that while this strategy provides strong excited-state reductants with red-shifted absorption profiles, it prevents the complexes from being potent excited state oxidants. Accordingly, the E*^/–^ values tabulated in Table 5, show that complexes **2** and **3** are in fact poor oxidants. This result is again a direct result of the high-energy donor HOMO of the azanidophenolate ligand.

Charge-transfer complexes with properties like **1–3** are ideal candidates for incorporation into dye-sensitized solar cells (DSSCs), where binding of the dye to the TiO_2_ surface provides a fast, intramolecular pathway for charge injection. In DSSCs incorporating [Ru(bpy)_3_]^2+^-type dyes, charge injection from the bpy π* orbital into the conduction band of TiO_2_ occurs on the sub-picosecond timescale.^65,66^ Given that **1–3** use the same bpy π* acceptor orbital, similarly rapid charge-injection rates should be possible for these dyes. Furthermore, pairing a low-potential “red” dye like **2** or **3** with a high-potential “blue” dye (*i.e.*, a hole injecting dye) could lead to highly efficient, tandem, photoelectrochemical cells for water-splitting applications.^67–73^ Importantly, the charge-transfer complexes reported here do not rely on scarce and precious metals like ruthenium, iridium, or platinum. To further develop this family of dyes, derivatives of **2** and **3** must be prepared that incorporate carboxylate or phosphonate linkers capable of binding to nanocrystalline TiO_2_. The excited-state dynamics of these donor–acceptor LL'CT dyes must be examined to derive lifetimes for 2* and 3* and to measure the rates and overall efficiency of electron injection into the semiconductor. These experiments are currently underway.
