# Supplementary material for: Near-IR absorbing donor–acceptor ligand-to-ligand charge-transfer complexes of nickel(ii)
Source: Chem Sci. 2015 Dec 8;7(3):1807–14. doi: 10.1039/c5sc02703a (PMC5604403; doi:10.1039/c5sc02703a)
Supplement: Supplementary file 1 [file SC-007-C5SC02703A-s001.pdf]

Electronic Supplementary Material (ESI) for Chemical Science.
This journal is © The Royal Society of Chemistry 2015

Near-IR Absorbing Donor-Acceptor Ligand-to-Ligand Charge-Transfer Complexes of Nickel(II)

L. A. Cameron, J. W. Ziller and A. F. Heyduk*

Department of Chemistry, University of California, Irvine, California, 92697

-- Supporting Information--

Experimental Procedures	S2-S5
^{1}H and ^{13}C NMR Spectra for complexes **1-3**	S6-S8
Variable-temperature ^{1}H NMR data for complexes **2** and **3**	S9-S10
Electrochemical analysis for complexes **1-3**	S11-S13
Solvatochromism data for complexes **1-3**	S14-S15
DFT data for complexes **1-3**	S16-S21

General Considerations. All compounds and reactions reported below show various levels of air- and moisture-sensitivity, so all manipulations were carried out using standard vacuum-line, Schlenk-line and glovebox techniques. Solvents were sparged with argon before being deoxygenated and dried by passage through Q5 and activated alumina columns, respectively. To test for effective oxygen and water removal, aliquots of each solvent were treated with a few drops of a purple solution of sodium benzophenone ketyl radical in THF. The reagents $Ni(cod)_2$ (Strem), 3,5-di-*tert*-butyl-1,2-quinone (Aldrich), and 4,4'-di-*tert*-butyl-2,2'-bipyridine (Aldrich) were reagent grade or better and used as received. The iminoquinones, 3,5-di-*tert*-butyl(2,6-diisopropylphenyl)-*ortho*-iminoquinone [1] and 9,10-(2,6-diisopropylphenyl)iminophenanthrenquinone [2] were prepared according to literature procedures. Elemental analyses were performed on a PerkinElmer series II 2400 CHNS analyzer.

Spectroscopic Measurements. NMR spectra were collected at 298 K on a Bruker Avance 400 MHz or 500 MHz spectrometer in dry, degassed C_6D_6 or $CDCl_3$. ^{1}H and ^{13}C NMR spectra were referenced to tetramethylsilane (TMS) using the residual ^{1}H and ^{13}C impurities of the deuterated solvent.[3] All chemical shifts are reported using the standard δ notation in parts per million; positive chemical shifts are to a higher frequency of TMS. Electronic absorption spectra were recorded with a PerkinElmer Lamda 900 UV-vis-NIR Spectrometer using one-centimeter path-length cells at ambient temperature (20-24 °C).

Electrochemical Methods. Electrochemical experiments were recorded on a Gamry Series G300 potentiostat/galvanostat/ZRA (Gamry Instruments, Warminster, PA) using a 3.0 mm glassy carbon working electrode, a platinum wire auxiliary electrode, and a silver wire pseudo-reference electrode. Reversibility of a redox process was judged based on the ratio of the anodic to the cathodic current being close to unity ($i_{pa}/i_{pc} \cong 1$) for a given process. Electrochemical experiments were performed at ambient temperature (20-24 °C) in a nitrogen-filled glovebox using THF solutions containing 1 mM analyte and 100 mM $[NBu_4][PF_6]$ as the supporting electrolyte. All potentials are referenced to $[Cp_2Fe]^{+/0}$ using ferrocene or decamethylferrocene (−0.49 V vs $[Cp_2Fe]^{+/0}$)[4] as internal standards. Ferrocene and decamethylferrocene (Acros) were purified by sublimation under reduced pressure and tetrabutylammonium hexafluorophosphate (Acros) was recrystallized from ethanol three times and dried under vacuum.

X-ray Data Collection and Reduction. X-ray diffraction data for all complexes were collected on single crystals mounted on either a glass fiber or a cryoloop and coated with oil. Data were acquired using a Bruker SMART APEX II diffractometer at 143 K using Mo Kα radiation (λ = 0.71073 Å) . The APEX2[5] program package was used to determine unit-cell parameters and for data collection. The raw frame data were processed using SAINT[6] and SADABS[7] to yield the reflection data file. Subsequent refinement cycles were carried out using the SHELXTL program suite.[8] Analytical scattering factors for neutral atoms were used throughout the analyses.[9] ORTEP diagrams were generated using ORTEP-3 for Windows.[10] Diffraction data for **1**, **2**, and **3** are given in Table S1.

Density Functional Theory Computations. Calculations were performed in the Molecular Modeling Facility in the Department of Chemistry at UC Irvine. Calculations

were performed employing Meta-GGA functional TPSS.[11] Geometry optimizations were initiated using a split-valence plus polarization basis set (def2-SVP)[12] and further refined using the polarized triple-ζ basis set def2-TZVP.[13] Structures obtained from single-crystal X-ray diffraction experiments were used as the starting points for geometry optimizations; no molecular symmetry was imposed. For complexes **1** and **3**, molecular geometries and orbital energies were evaluated self-consistently to tight convergence criteria (energy converged to 0.1 μHartree, maximum norm of the Cartesian gradient ≤ 10^{-4} a.u.). For complex **2,** ultra tight convergence criteria (0.01 μHartree, maximum norm of the Cartesian gradient ≤ 10^{-6} a.u.) yielded a single imaginary frequency (–6 cm^{-1}) that was identified as numerical noise upon vibrational analysis. Mulliken population analyses were obtained at TPSS/TZVP theory level; the contour values were 0.03 for the molecular orbital plots. All calculations were performed using the quantum chemistry program package TURBOMOLE.[14,15]

Table S1. X-ray diffraction data collection and refinement parameters for (cat)Ni(bpy^{t}Bu$_2$) (**1**), (ap)Ni(bpy^{t}Bu$_2$) • 2 (C_6H_6) (**2** • 2 (C_6H_6)), and (ap^{Ph})Ni(bpy^{t}Bu$_2$) • $C_4H_{10}O$ • C_6H_6 (**3** • $C_4H_{10}O$ • C_6H_6).

	**1**	**2** • 2 (C_6H_6)	**3** • Et_2O • C_6H_6
empirical formula	$C_{32}H_{44}N_2NiO_2$	$C_{56}H_{73}N_3NiO$	$C_{54}H_{65}N_3NiO_2$
formula weight	547.40	862.88	846.80
crystal system	Monoclinic	Orthorhombic	Monoclinic
space group	$P2_1/c$	$P2_12_12_1$	$P2_1/c$
a / Å	10.2568(9)	15.7714(10)	10.3735(5)
b / Å	19.4457(18)	17.0164(11)	18.2520(8)
c / Å	15.3511(14)	18.6084(12)	24.6774(11)
α / deg	90	90	90
β / deg	107.5750(12)	90	97.1921(6)
γ / deg	90	90	90
V / Å^3	2918.9(5)	4994.0(6)	4635.6(4)
Z	4	4	4
refl. collected	35094	44108	55375
indep. refl.	7204 [R(int) = 0.0419]	11901 [R(int) = 0.0449]	11708 [R(int) = 0.0203]
R1 ($I > 2\sigma$)^a	0.0337	0.0387	0.0312
wR2 (all data)^b	0.0794	0.0839	0.0795

^{a}R1 = $\Sigma||F_o|-|F_c||/\Sigma|F_o|$; ^{b}$w$R2 = $[\Sigma[w(F_o^2-F_c^2)^2]/\Sigma[w(F_o^2)^2]]^{1/2}$

Synthesis of (cat)Ni(bpy^{t}Bu$_2$) (1). A benzene solution of a Ni(cod)$_2$ (138 mg, 0.50 mmol, 1.0 equiv) and 3,5-bis(*tert*-butyl)-1,2-benzenedione (110 mg, 0.50 mmol, 1.0 equiv) was stirred at ambient glovebox temperature for one hour. Solid 4,4'-di-*tert*-butyl-2,2'-bipyridine (134 mg, 0.50 mmol, 1.0 equiv) was then added to the solution and stirring was continued for 2 days. The volume of the resulting dark blue solution was reduced and pentane was added to yield the product as a blue crystalline solid in 80% yield (219 mg).

X-ray quality crystals were grown by vapor diffusion of diethyl ether into a solution of **1** in benzene.

^{1}H NMR (400 MHz, $CDCl_3$) δ/ppm: 1.26 (*s*, 9H), 1.40 (*s*, 18H), 1.47 (*s*, 9H), 6.34 (*d*, *J* = 2.1 Hz, 1H), 6.55 (*d*, *J* = 2.2 Hz, 1H), 7.36 (*m,* 1H), 7.47 (*d, J= 5.7* Hz, 1H), 7.48, 7.65 (*s*, 2H), 8.63 (*d*, *J* = 5.7 Hz, 1H), 8.71 (*d*, *J* = 5.7 Hz, 1H). ^{13}C NMR (126 MHz, $CDCl_3$,) δ/ppm: 163.3 (*C*=N), 160.9 (*C*–O), 160.7 (*C*–O), 156.9 (aryl–*C*), 153.1 (aryl–*C*), 149.7 (aryl–*C*), 149.5 (aryl–*C*), 136.6 (aryl–*C*H), 133.0 (aryl–*C*H), 123.0 (aryl–*C*H), 122.6 (aryl–*C*H), 120.6 (aryl–*C*H), 118.2 (aryl–*C*H), 116.6 (aryl–*C*H), 109.5 (aryl–*C*H), 109.2 (aryl–*C*H), 35.5 (*C*–$(CH_3)_3$) , 34.2 (*C*–$(CH_3)_3$), 33.9 (*C*–$(CH_3)_3$), 32.1 ((–$CH_3)_3$), 30.6 ((–*C*$H_3)_3$), 30.3 ((–*C*$H_3)_3$).

Anal. Calcd. for $C_{32}H_{44}N_2O_2Ni$: C, 70.21; H, 8.10; N 5.12. Found: C, 70.09; H, 8.15; N, 5.05 %. UV-vis-NIR (THF) λ_{max}/nm (ε/M^{-1} cm^{-1}): 370 (2000), 620 (3600)

Synthesis of (ap)Ni(bpy^{t}Bu$_2$) (2). A benzene solution of a Ni(cod)$_2$ (138 mg, 0.50 mmol, 1.0 equiv) and 3,5-di-*tert*-butyl(2,6-diisopropylphenyl)-*ortho*-iminoquinone (190 mg, 0.50 mmol, 1.0 equiv) was stirred at ambient glovebox temperature for one hour. Solid 4,4'-di-*tert*-butyl-2,2'-bipyridine (134 mg, 0.50 mmol, 1.0 equiv) was then added to the solution and stirring was continued for 2 days. The solvent was stripped from the resulting dark yellow-green solution and the solid residue was redissolved in toluene, diluted with pentane, and chilled to −35 °C. The precipitated solid was collected by filtration, washed with cold pentane, and dried under reduced pressure to obtain the desired product as a black crystalline solid in 66% yield (215 mg). X-ray quality crystals were obtained from concentrated solution of **2** in benzene.

^{1}H NMR (500 MHz, C_6D_6) δ/ppm: 0.72 (br *s*, 9H), 0.91 (br *s*, 9H), 1.26 (*d*, *J* = 6.9 Hz, 6H), 1.30 (*d*, *J* = 6.9 Hz, 6H), 1.51 (*s*, 9H), 2.01 (*s*, 9H), 4.59 (*m*, 2H), 6.27 (*d*, *J* = 2.1 Hz, 1H), 6.59 (*s*, 1H,), 6.88 (br *s*, 2H), 6.95 (*d*, *J* = 2.1 Hz, 1H), 7.14 (*d*, *J* = 1.6 Hz, 2H), 7.42 (*d, J* = 6.7, 2H), 7.48 (*m*, 1H), 9.79 (*s*, 1H). ^{13}C NMR (101 MHz, C_6D_6,) δ/ppm: 160.9 (N=*C*), 160.7 (aryl–*C*), 155.9 (*C*–O), 155.1 (*C*–N), 154.2 (aryl–*C*), 152.1 (aryl–*C*), 150.1 (br, N–*C*H), 149.9 (aryl–*C*(ipp), 147.7 (aryl–*C*), 137.2 (aryl–*C*), 131.5 (aryl–*C*), 128.4 (aryl–*C*H, 125.8 (diip aryl–*C*H), 125.1 (diip aryl–*C*H), 123.5 (aryl–*C*), 123.4 (aryl–*C*), 122.2 (aryl–*C*), 121.4 (aryl–*C*), 116.6 (aryl–*C*), 116.1 (aryl–*C*), 109.2 (aryl–*C*H), 108.5 (aryl–*C*H), 35.1 (ap–*C*(CH3)3), 34.7 (*C*$(CH_3)_3$), 32.8 (C(*C*$H_3)_3$), 31.0 (C(*C*$H_3)_3$), 29.9 (br, C(*C*$H_3)_3$), 29.6 (br, C(*C*$H_3)_3$), 28.2 (*C*H$(CH_3)_2$), 24.9 (CH(*C*$H_3)_2$), 24.4 (CH(*C*$H_3)_2$).

Anal. Calcd. for $C_{44}H_{61}N_3ONi$: C, 74.78; H, 8.70; N, 5.95. Found: C, 74.52; H, 8.60; N, 5.41%. UV-vis-NIR (THF) λ_{max}/nm (ε/M^{-1} cm^{-1})]: 310 (19600), 890 (6200).

Synthesis of (ap^{ph})Ni(bpy^{t}Bu$_2$) (3). The complex (ap^{ph})Ni(bpy^{t}Bu$_2$) by the same method used to prepare 2, using 134 mg of Ni(cod)$_2$ (0.5 mmol, 1 equiv), 134 mg of 4,4'-di-*tert*-butyl-2,2'-bipyridine (134 mg, 0.5 mmol), and 180 mg of 9,10-(2,6-diisopropylphenyl)iminophenanthrenquinone (0.5 mmol, 1 equiv). The product was isolated as black crystals in 81% yield (260 mg). X-ray quality crystals were grown by vapor diffusion of diethyl ether into a solution of **3** dissolved in benzene.

^{1}H NMR 500 MHz (C_6D_6) δ/ppm: 0.86 (*s,* 18H), 1.14 (*d*, *J* = 6.9 Hz, 6H) 1.31 (*d*, *J* = 6.9 Hz 6H,) 4.76 (*m*, 2H), 6.59 (*d*, *J* = 5.4 Hz, 2H,), 7.12 (*d*, *J* = 1.6 Hz, 2H), 7.18 (*m*, 2H,), 7.25 (*t*, *J* = 7.4 Hz, 1H), 7.37 (*d*, *J* = 7.7 Hz, 2H), 7.47 (*t*, *J* = 7.5 Hz, 1H), 7.55 (*t*, *J* = 7.6 Hz, 1H), 7.63 (*d*, *J* = 8.6 Hz, 1H), 7.83 (*t*, *J* = 7.4 Hz, 1H), 8.73 (*d*, *J* = 8.3 Hz, 1H),

8.79 (*d*, *J* = 8.2 Hz, 1H,), 9.16 (*d*, *J* = 8.1 Hz, 1H). ^{13}C NMR (126 MHz; C_6D_6) δ/ppm: 160.5 (N=*C*), 152.3 (*C*–O), 149.6 (aryl–*C*), 147.8 (aryl–*C*), 141.1 (aryl–*C*), 129.22 (aryl–*C*), 129.0 (aryl–*C*), 128.5 (aryl–*C*), 126.5 (aryl–*C*), 125.6 (aryl–*C*H), 125.2 (aryl–*C*H), 124.4 (aryl–*C*H), 124.1 (aryl–*C*H), 123.5 (aryl–*C*H), 123.4 (aryl–*C*H), 122.7 (aryl–*C*H), 121.1 (aryl–*C*H), 121.0 (aryl–*C*H), 120.6 (aryl–*C*H), 116.2 (aryl–*C*H), 34.7, 30.5 (–C(*C*H$_3$)$_3$), 29.8 (–C(*C*H$_3$)$_3$), 28.6 (*C*H(CH$_3$)$_2$), 23.9 (CH(*C*H$_3$)$_2$), 23.8 (CH(*C*H$_3$)$_2$).

Anal. calcd for $C_{44}H_{49}N_3ONi \bullet C_6H_6$: C, 77.72; H, 7.17; N, 5.44. Found: C, 77.43; H, 7.09; N, 5.24%. UV-vis-NIR (THF) λ_{max}/nm (ε/M^{-1} m^{-1}): 400 (18000), 955 (8100).

References

1. G. A. Abakumov, *Russ. Chem. Bull.,* 2003, **52**, 712.
2. G. A. Abakumov, V. K. Cherkasov, N. O. Druzhkov, Y. A. Kurskii, G. K. Fukin, L. G. Abakumova, and T. N. Kocherova, *Synth. Comm.*, 2006, **36**, 3241.
3. H. Gottlieb, V. Kotlyar, A. Nudelman, *J. Org. Chem.*, 1999, **62**, 7512.
4. N. G. Connelly, and W. E. Geiger, *Chem. Rev.,* 1996, **96**, 877.
5. APEX2 Version 2013.6-2, Bruker AXS, Inc.; Madison, WI 2013.
6. SAINT Version 8.32b, Bruker AXS, Inc.; Madison, WI 2012.
7. Sheldrick, G. M. SADABS, Version 2012/1, Bruker AXS, Inc.; Madison, WI 2012.
8. Sheldrick, G. M. SHELXTL, Version 2014/7, Bruker AXS, Inc.; Madison, WI 2014.
9. *International Tables for Crystallography*, A. J. C. Wilson, Ed.; Kluwer Academic, Dordrecht, Netherlands, 1992, Vol. C.
10. L. J. Farrugia, *J. Appl. Crystallogr.*, 1997, **30**, 565.
11. J. Tao, J. P. Perdew, V. N. Staroverov, and G. E. Scuseria, *Phys. Rev. Lett.*, 2003**, 91**, 146401.
12. A. Schäfer, H. Horn, and R. Ahlrichs, *J. Chem. Phys*., 1992**, 97**, 2571.
13. A. Schäfer, C. Huber and R. Ahlrichs, *J. Chem. Phys.,* 1994, **100**, 5829.
14. TURBOMOLE V6.3, Turbomole GmbH, Karlsruhe, 2011. http://www.turbomole.com.
15. F. Furche, R. Alrichs, C. Hättig, W. Klopper, M. Sierka and F. Weigend, *WIREs Comp. Mol. Chem*., **2014**, *4*, 91.

^{1}H and ^{13}C NMR spectra for complexes 1-3.

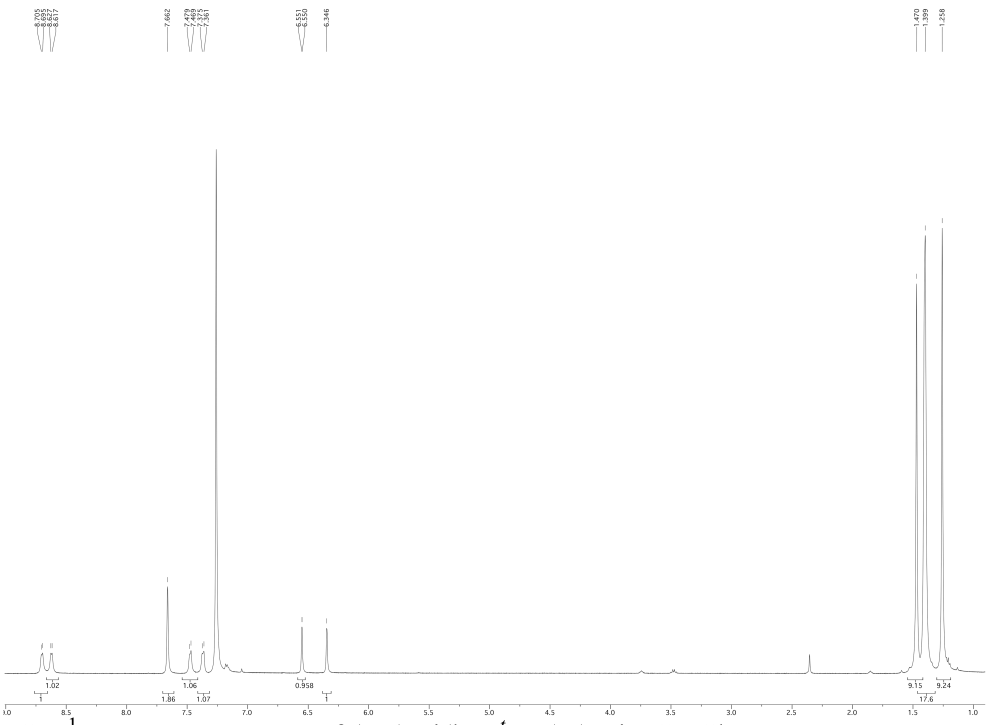

Figure S1. ^{1}H NMR spectrum of (cat)Ni(bpy^{t}Bu$_2$) (**1**) in $CDCl_3$.

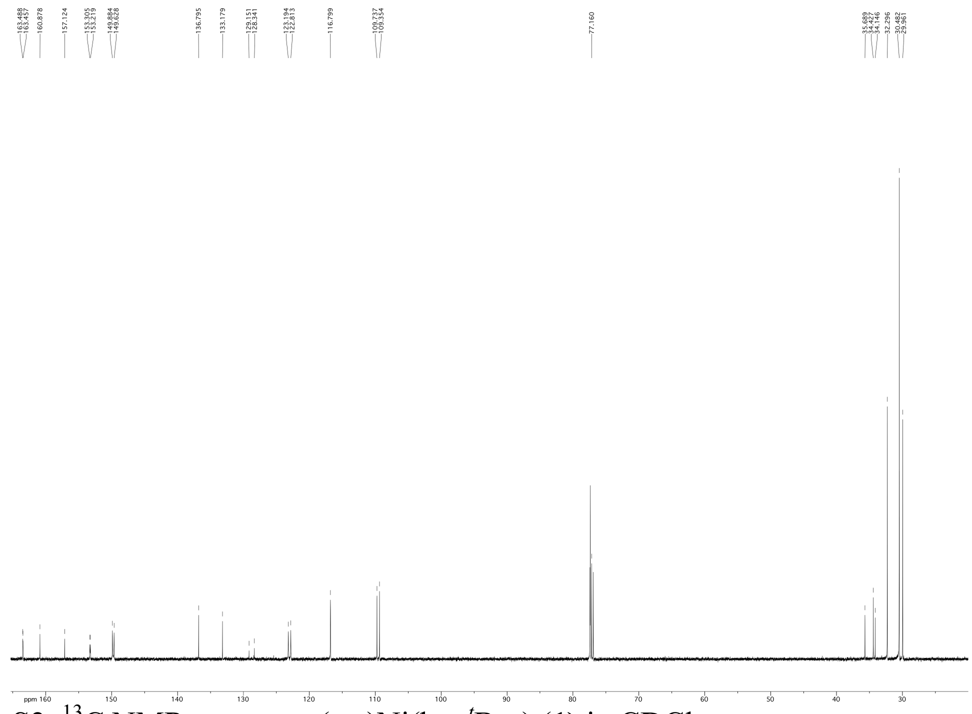

Figure S2. ^{13}C NMR spectrum (cat)Ni(bpy^{t}Bu$_2$) (**1**) in $CDCl_3$.

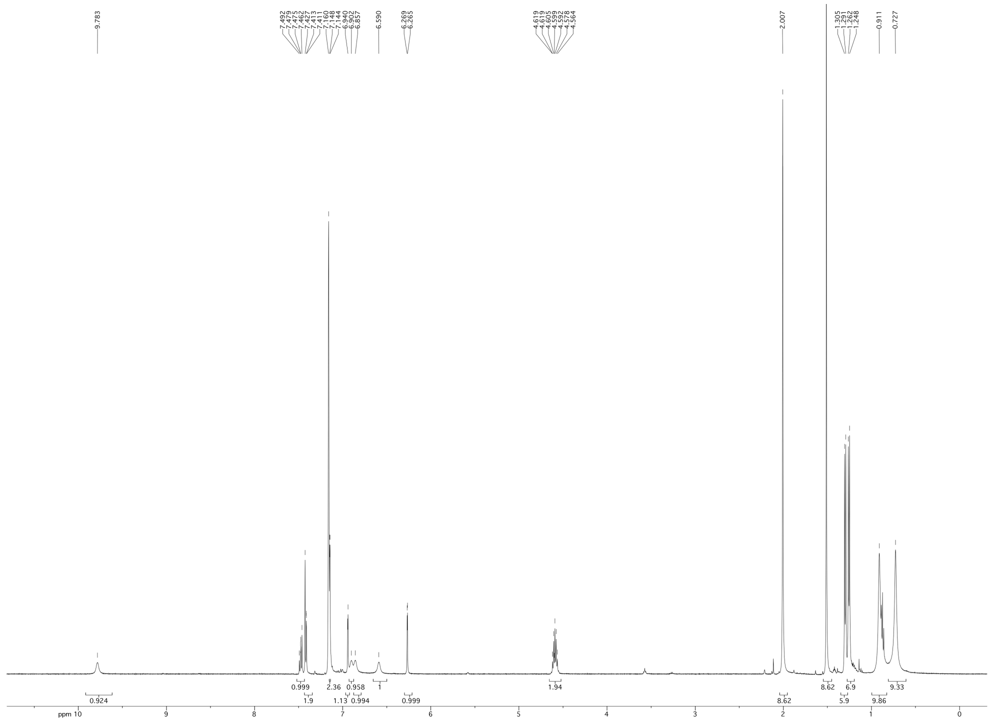

Figure S3. ^{1}H NMR spectrum of (ap)Ni(bpy^{t}Bu$_2$) (**2**) in C_6D_6.

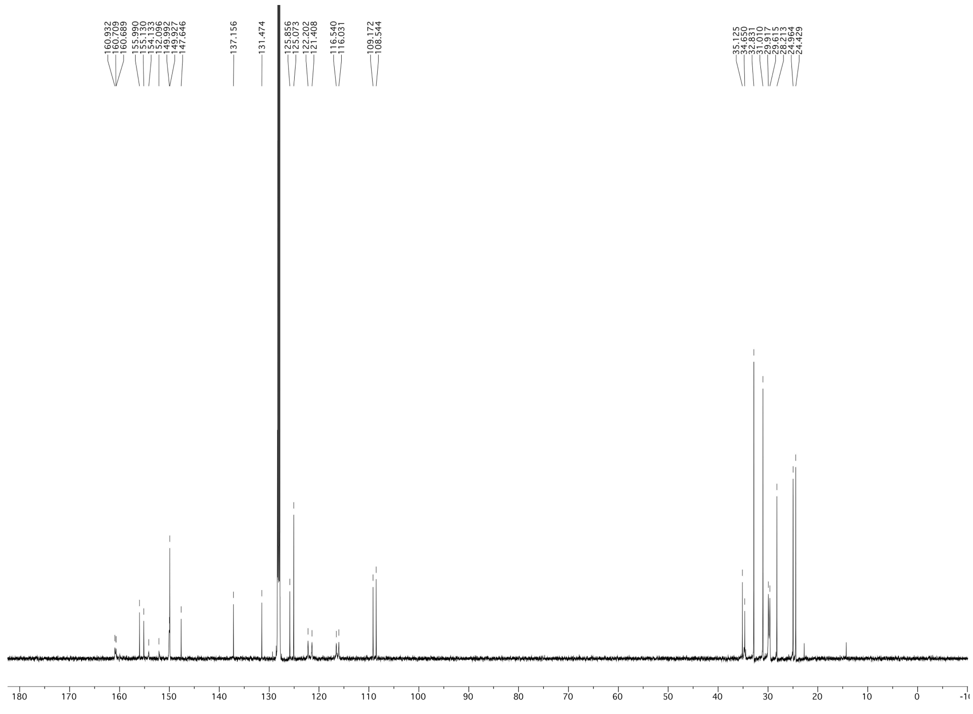

Figure S4. ^{13}C NMR spectrum (ap)Ni(bpy^{t}Bu$_2$) (**2**) in C_6D_6.

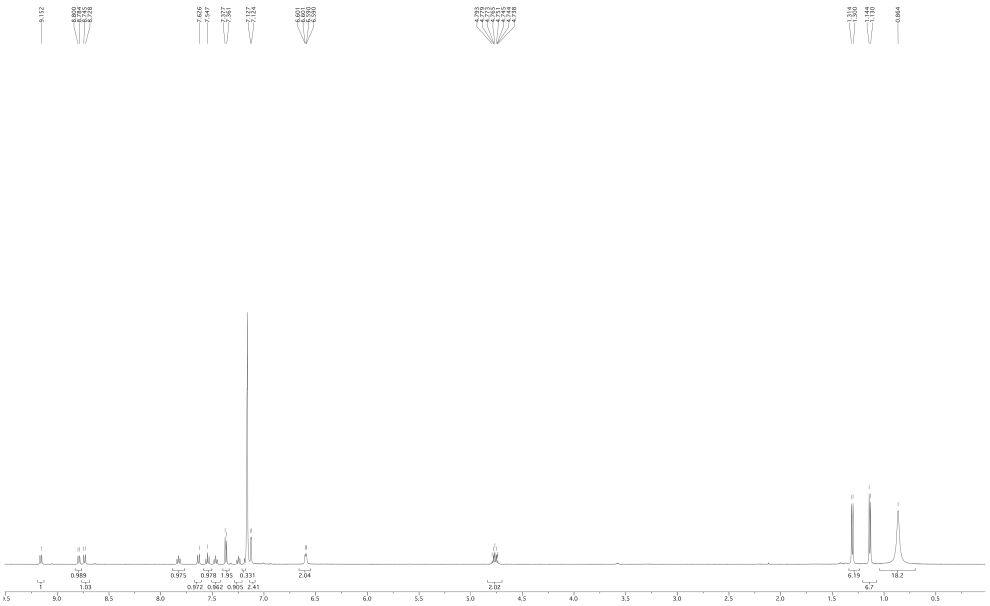

Figure S5. ^{1}H NMR spectrum of (ap^{Ph})Ni(bpy^{t}Bu$_2$) **(3)** in C_6D_6.

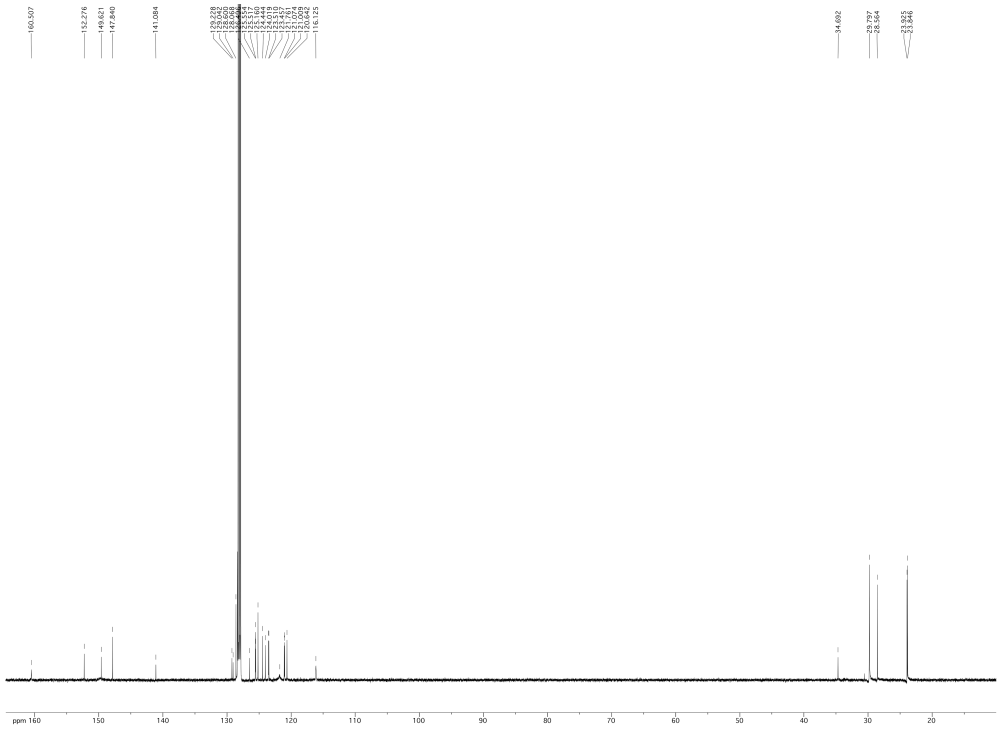

Figure S6. ^{13}C NMR spectrum (**3**) in C_6D_6.

Variable-temperature ^{1}H NMR data for 2 and 3.

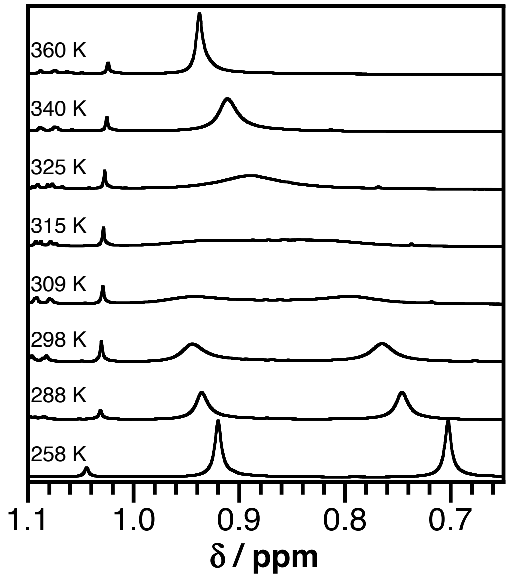

Figure S7. Partial ^{1}H NMR spectra (500 MHz) of (ap)Ni(bpy^{t}Bu$_2$) (**2**) in toluene-d_8 showing the *tert*-butyl proton resonances of the bpy^{t}Bu$_2$ ligand over the temperature range 258-360 K.

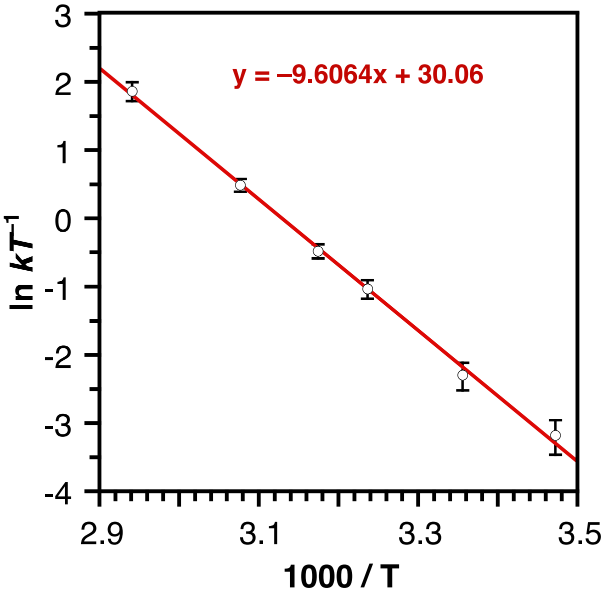

Figure S8. Eyring plot for (ap)Ni(bpy^{t}Bu$_2$) (**2**).

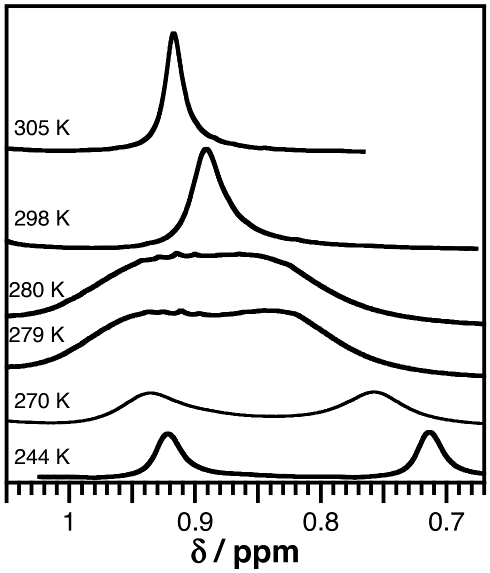

Figure S9. Partial ^{1}H NMR spectra (500 MHz) of $(ap^{Ph})Ni(bpy^{t}Bu_2)$ (**3**) in toluene-d_8 showing the *tert*-butyl proton resonances of the $bpy^{t}Bu_2$ ligand over the temperature range 244-305 K.

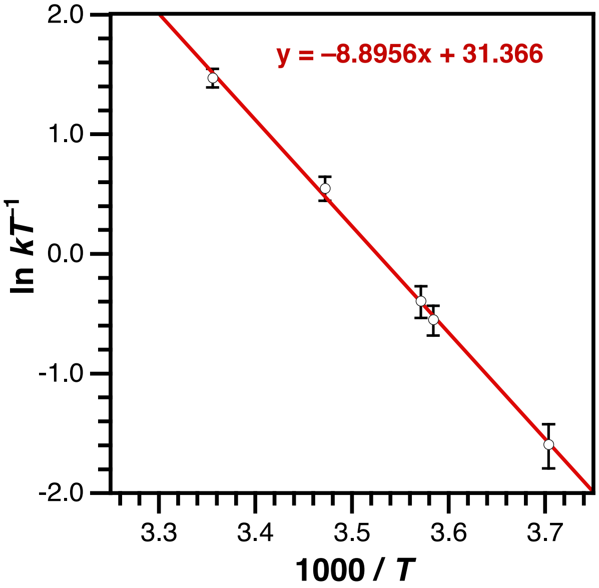

Figure S10. [1]H Eyring Plot of $(ap^{Ph})Ni(bpy^{t}Bu_2)$ (**3**).

Electrochemical analysis of complexes 1-3.
Data were collected at 298 K in THF solutions that were 1.0 mM in analyte and 0.1 M in $[Bu_4N][PF_6]$ electrolyte using a glassy carbon working electrode, a platinum wire counter electrode and a silver wire pseudo-reference electrode. All potentials were referenced to $[Cp_2Fe]^{+/0}$ using an internal standard that was added at the end of the experiment.

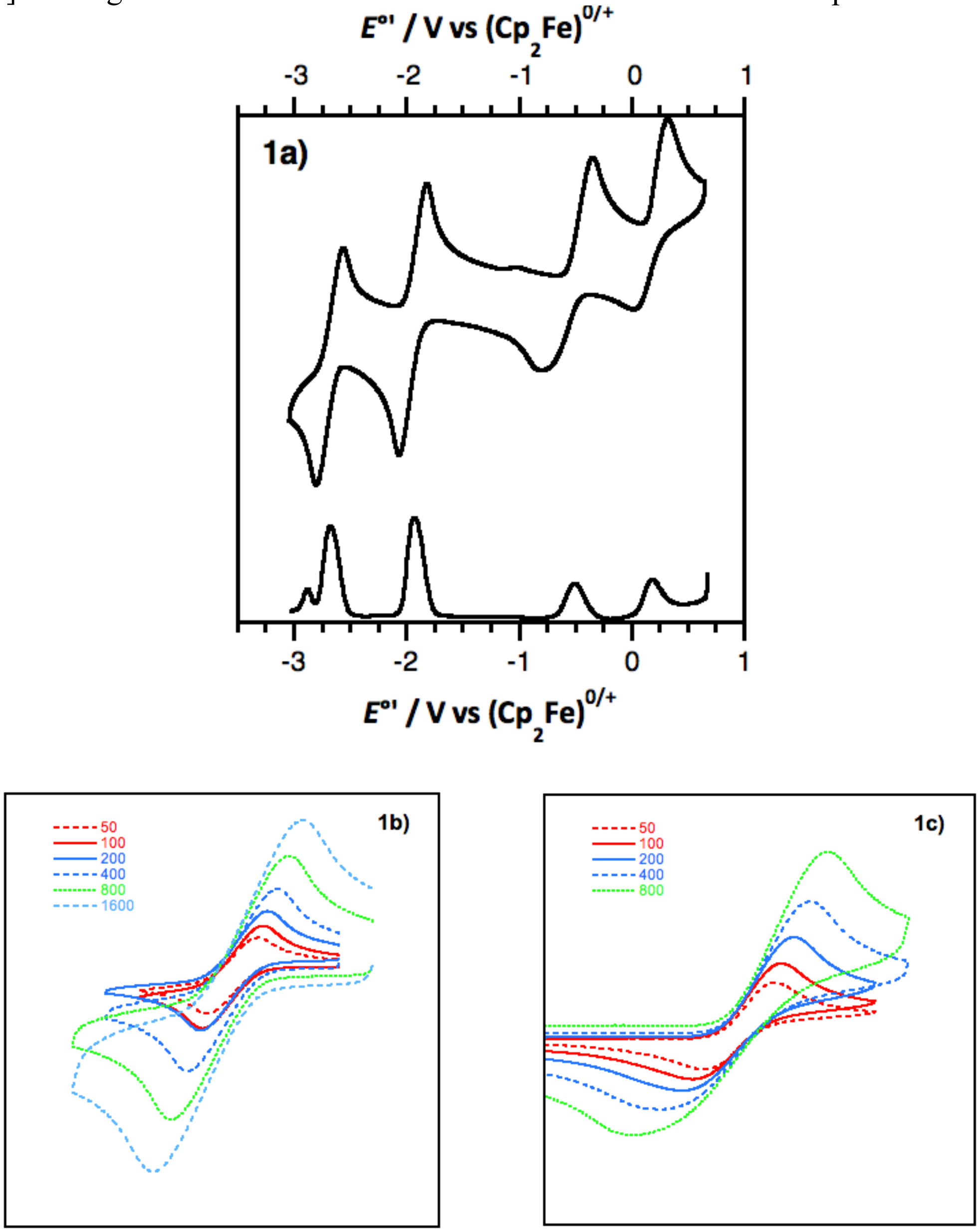

Figure S11. (a) Cyclic voltammogram and differential pulse voltammogram for (cat)Ni(bpy^{t}Bu$_2$) (**1**) in THF, (b) scan-rate dependence of the first reduction ($E°'_3$) of **1**, and (c) scan-rate dependence of the first oxidation ($E°'_2$) of **1**.

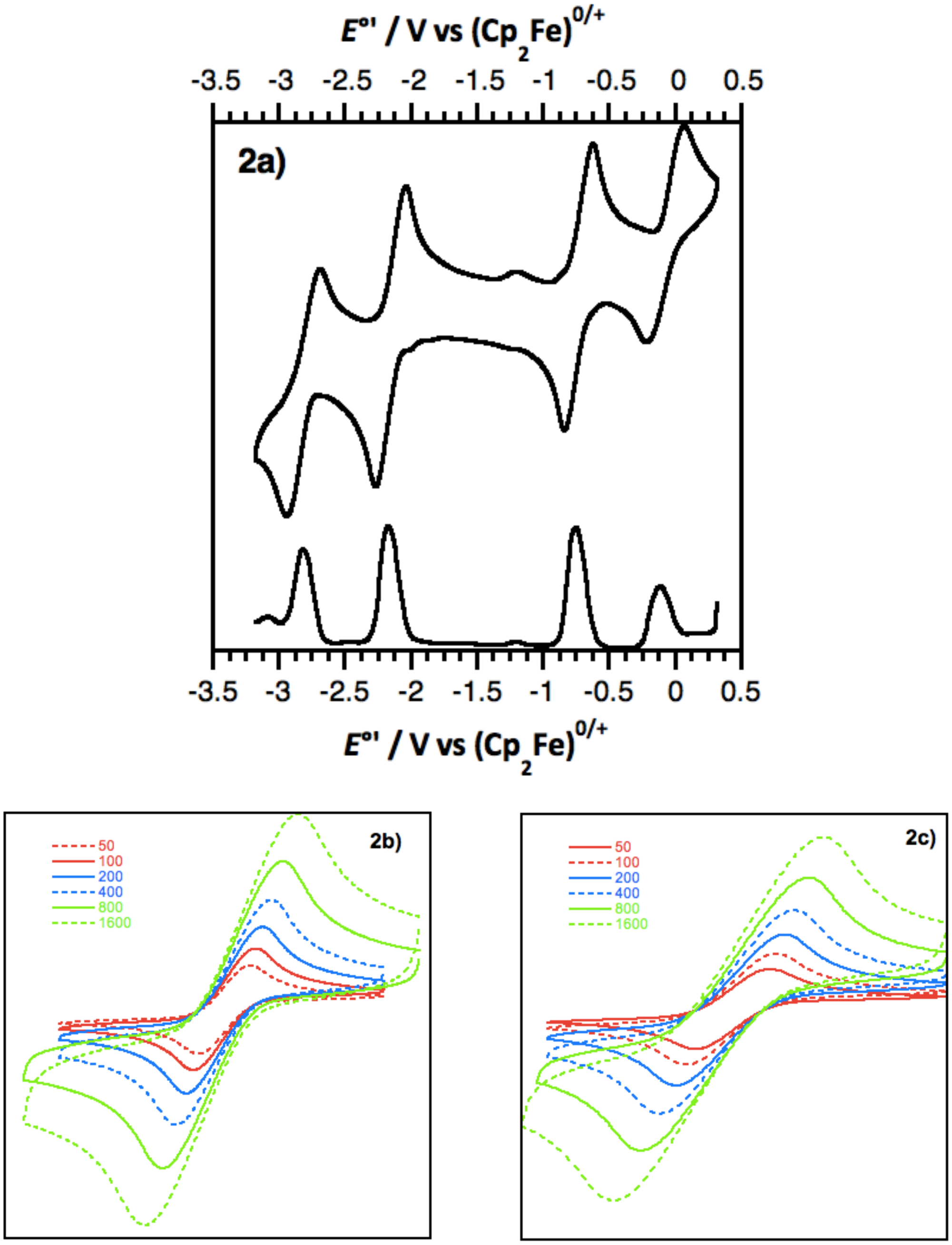

Figure S12. (a) Cyclic voltammogram and differential pulse voltammogram for (ap)Ni(bpy^{t}Bu$_2$) (**2**) in THF, (b) scan-rate dependence of the first reduction ($E°'_3$) of **2**, and (c) scan-rate dependence of the first oxidation ($E°'_2$) of **2**.

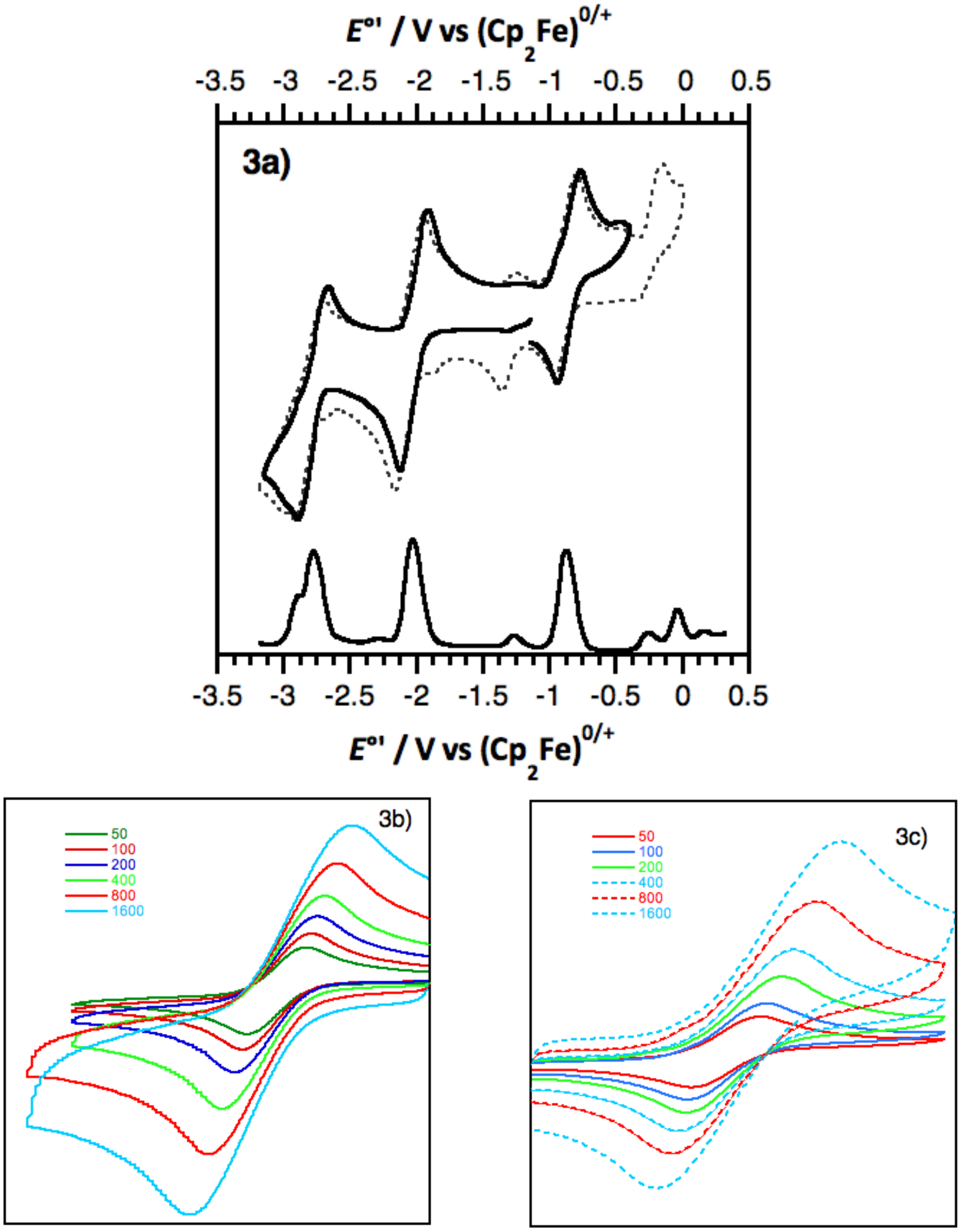

Figure S13. (a) Cyclic voltammogram and differential pulse voltammogram for (ap^{Ph})Ni(bpy^{t}Bu$_2$) (**2**) in THF, (b) scan-rate dependence of the first reduction ($E°'_3$) of **2**, and (c) scan-rate dependence of the first oxidation ($E°'_2$) of **2**.

Solvent-Dependence of the LL'CT Transition in complexes 1-3.

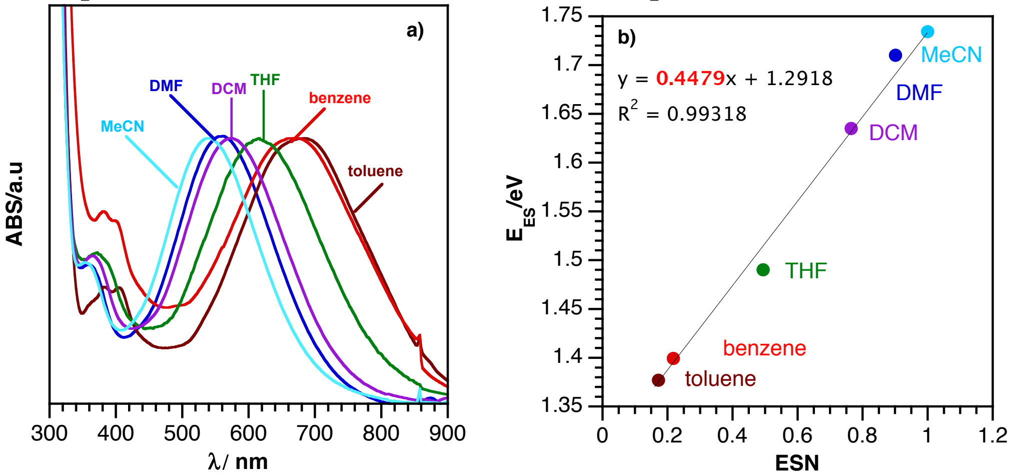

Figure S14. (a) UV-vis-NIR absorption spectra of (cat)Ni(bpy^{t}Bu$_2$) (**1**) in various solvents and (b) plot of the estimated excited state energy for **1** vs. the effective solvent number.

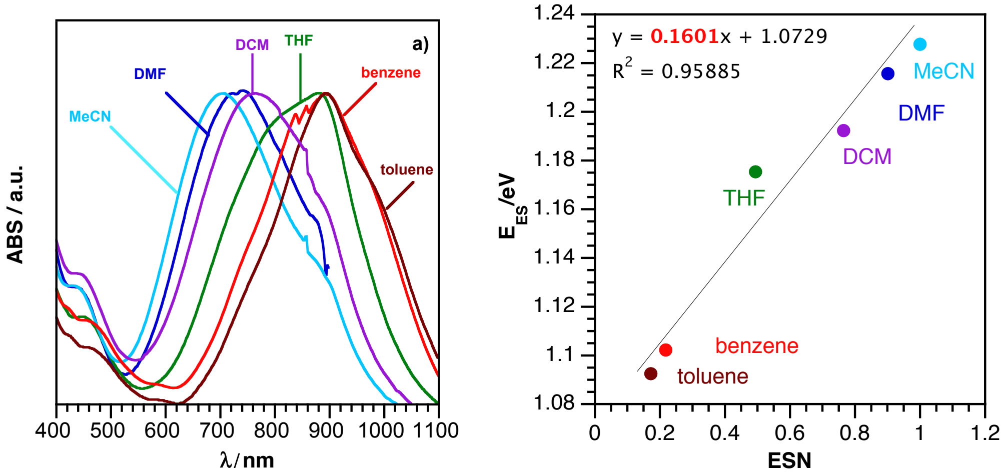

Figure S15. (a) UV-vis-NIR absorption spectra of (ap)Ni(bpy^{t}Bu$_2$) (**2**) in various solvents and (b) plot of the estimated excited state energy for **2** vs. the effective solvent number.

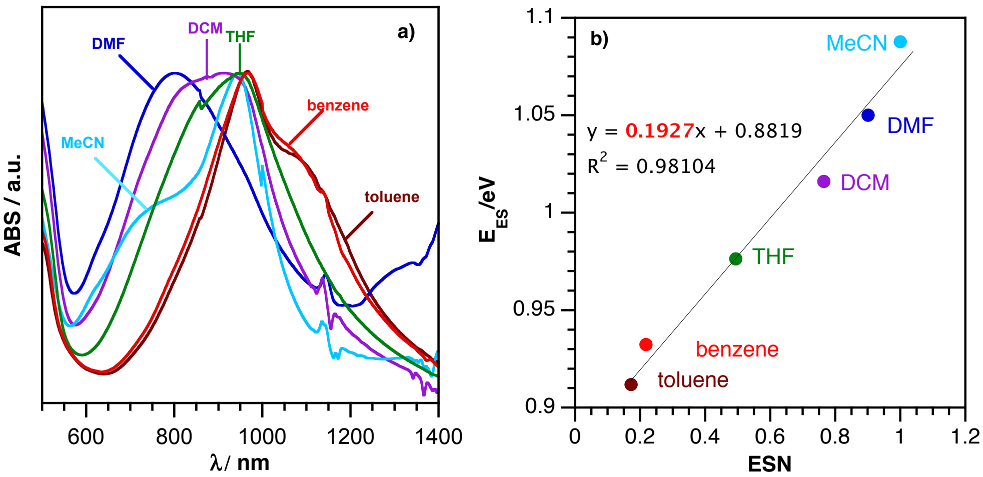

Figure S16. (a) UV-vis-NIR absorption spectra of (ap^{Ph})Ni(bpy^{t}Bu$_2$) (**3**) in various solvents and (b) plot of the estimated excited state energy for **3** vs. the effective solvent number.

Density Functional Theory

Table S2a. Orbital contributions and calculated energies for (cat)Ni(bpy^{t}Bu$_2$) (**1**) as determined by TPSS/def2-TZVP DFT computations.

1 (cat)Ni(bpy^{t}Bu$_2$)

	% Contribution			
**Orbital**	**Ni**	**Donor**	**Acceptor**	**Energy / eV**
LUMO+4	0.0	0.0	100.0	-0.662
LUMO+3	1.9	0.0	98.1	-1.849
LUMO+2	2.2	0.0	97.8	-2.131
LUMO+1	59.5	24.2	16.3	-2.316
LUMO	8.1	10.9	80.9	-2.914
HOMO	5.8	79.1	15.0	-3.682
HOMO-1	31.8	68.2	0.0	-4.397
HOMO-2	93.2	6.8	0.0	-4.795
HOMO-3	54.8	41.8	3.4	-5.156
HOMO-4	71.8	19.6	12.0	-5.264

Table S2b. Optimized coordinates of (cat)Ni(bpy^{t}Bu$_2$) (**1**) in XYZ format.

				H	-4.7338735	-5.9736993	-0.9723075
Energy		-3015.191927		H	-4.8139754	-4.2071881	-1.1412968
Ni	0.1414926	0.2549575	-0.0692183	C	-2.9842316	-5.2518941	-2.9309155
O	1.0067443	1.8391151	0.1458509	H	-3.5798726	-4.4486376	-3.3793771
O	0.1213628	0.0737855	1.744982	H	-3.5075309	-6.1958744	-3.1156594
N	0.1315556	0.3831183	-1.9314891	H	-2.0188368	-5.3007882	-3.4474769
N	-0.7478801	-1.3592364	-0.3751544	C	-1.9978475	-6.2447195	-0.8525583
C	-0.4639949	-0.6599488	-2.5955671	H	-1.0024539	-6.2900447	-1.3070302
C	-0.9754691	-1.6718437	-1.6891182	H	-2.5189098	-7.1842307	-1.0689106
C	-1.1754134	-2.2191269	0.575215	H	-1.8729169	-6.1702737	0.2322639
H	-0.9644563	-1.9134791	1.59407	C	-1.6356031	-2.8528938	-2.0502909
C	-1.8302881	-3.3943971	0.2626513	H	-1.7927331	-3.0562982	-3.1033484
H	-2.1462637	-4.0381031	1.0771632	C	-0.5334997	-0.6779455	-3.9897955
C	-2.0819638	-3.7479156	-1.0819201	H	-1.0133442	-1.5221287	-4.475264
C	-2.8093884	-5.0526677	-1.4152737	C	0.0000671	0.3632495	-4.7521527
C	-4.2099895	-5.0356938	-0.7562233	C	0.6034325	1.4162678	-4.037789
H	-4.1418465	-4.9297554	0.3309675	H	1.0424659	2.2635842	-4.5511104

C	0.651258	1.3954755	-2.6529971	H	4.4302529	5.1473191	5.126518
H	1.1060445	2.1858669	-2.0645868	H	4.6568836	3.7344549	4.0704261
C	-0.0842502	0.3281696	-6.282079	H	3.6508871	3.6035576	5.5221066
C	0.5615659	1.568009	-6.926083	C	3.1767252	5.6630136	2.7711728
H	0.4759599	1.4969741	-8.0155335	H	2.3398863	6.0291606	2.1658401
H	1.6263442	1.6447087	-6.6798455	H	3.9033412	5.1879587	2.1024163
H	0.0651712	2.4929253	-6.6124242	H	3.6623025	6.5306327	3.2329917
C	0.6450745	-0.9327092	-6.8050446	C	1.5137578	2.46316	4.1640948
H	0.1976773	-1.8500728	-6.4086087	H	1.6429515	2.6184291	5.2302535
H	1.7024755	-0.9196847	-6.5205381	C	0.8661704	1.2937207	3.7426555
H	0.5830897	-0.9724114	-7.8985643	C	0.3430881	0.2410628	4.7305404
C	-1.5702714	0.2702892	-6.7106766	C	-1.1874493	0.0834072	4.5614408
H	-2.1142228	1.152956	-6.3581643	H	-1.4432103	-0.1869045	3.5339882
H	-2.0701191	-0.6186211	-6.3119366	H	-1.6978328	1.0220563	4.8053136
H	-1.6416862	0.2354512	-7.8037906	H	-1.5633159	-0.6959833	5.2371211
C	1.2057561	2.1055275	1.4565978	C	0.6152682	0.6250885	6.1965289
C	1.8468667	3.2602304	1.9125752	H	0.2220516	-0.1603132	6.8528768
H	2.2033373	3.9800054	1.182935	H	0.1233373	1.565763	6.4688077
C	2.0076139	3.4506057	3.2877314	H	1.6877613	0.7240983	6.3985479
C	2.7002796	4.6948262	3.8689311	C	1.0388483	-1.1153952	4.4623061
C	1.7171623	5.458521	4.7876507	H	0.8826876	-1.4396853	3.4304311
H	2.2044709	6.3438888	5.2157302	H	0.6418459	-1.8833128	5.1390367
H	1.3718935	4.8286412	5.6137849	H	2.1178118	-1.032418	4.6351336
H	0.8370945	5.7873756	4.2240727	C	0.7187215	1.1320898	2.3478857
C	3.9341047	4.2667982	4.6985138				

Table S3a. Orbital contributions and calculated energies for (ap)Ni(bpy^{t}Bu$_2$) (**2**) as determined by TPSS/def2-TZVP DFT computations.

2 (ap)Ni(bpy^{t}Bu$_2$)

	% Contribution			
**Orbital**	**Ni**	**Donor**	**Acceptor**	**Energy / eV**
LUMO+4	0.0	86.5	13.5	-0.835
LUMO+3	1.6	0.0	98.4	-1.678
LUMO+2	2.0	0.0	98.0	-1.934
LUMO+1	56.1	28.4	15.5	-2.487
LUMO	8.2	14.4	77.4	-2.818
HOMO	7.18	74.23	18.59	-3.555
HOMO-1	30.6	69.4	0.0	-4.279
HOMO-2	95.61	3.78	0.60	-4.747
HOMO-3	58.3	38.2	3.5	-5.116
HOMO-4	70.0	23.5	6.4	-5.247

Table S3b Optimized coordinates of (ap)Ni(bpy^{t}Bu$_2$) (**2**) in XYZ format.

Energy	-3462.467038		
Ni	-0.3447134	0.0170355	-0.34922
O	-0.7323929	-1.7668011	-0.5243612
N	0.8910249	-0.5530408	0.9471739
N	-1.6444302	0.3739981	-1.6813887
N	-0.1476075	1.9250654	-0.3786297
C	-0.0318247	-2.5908186	0.2702492
C	0.893747	-1.9348366	1.1133184
C	1.6935281	-2.6755612	1.9970661
H	2.399875	-2.156556	2.6383091
C	1.5753015	-4.0696999	2.0452699
C	0.6468059	-4.6925253	1.1952144
H	0.5545857	-5.7705884	1.2316049
C	-0.1743225	-3.9925951	0.2949286
C	2.4586171	-4.8705517	3.0195804
C	2.1845473	-4.4123872	4.4714603
H	2.812645	-4.9747605	5.174095
H	1.1350257	-4.5777542	4.7389478
H	2.4037424	-3.3476267	4.6010803
C	3.9510311	-4.6263203	2.6939664
H	4.5898513	-5.1899895	3.3858131
H	4.2097002	-3.5663456	2.7837306
H	4.1827617	-4.9467458	1.6721927
C	2.1981609	-6.3861405	2.943348
H	1.1615931	-6.6320368	3.1996043
H	2.8512744	-6.9041398	3.65541
H	2.4099356	-6.7835233	1.9444421
C	-1.17772	-4.7141029	-0.617596
C	-0.8311703	-4.4355053	-2.1002781
H	-0.8047619	-3.3631609	-2.3078383
H	-1.5743556	-4.9055981	-2.7574866
H	0.1524378	-4.8518824	-2.3455436
C	-1.1597154	-6.2414194	-0.4189178
H	-0.1783733	-6.6706446	-0.6499637
H	-1.892604	-6.6992363	-1.0938684
H	-1.42778	-6.5219869	0.6058214
C	-2.6129089	-4.2234938	-0.308418

H	-2.6927475	-3.1385037	-0.4092346	H	-2.8699988	3.1250369	-3.2182353
H	-2.8928756	-4.489481	0.7171837	C	-1.8291721	1.6861749	-2.0174318
H	-3.3301546	-4.6966932	-0.9917088	C	-0.9726294	2.575217	-1.2714343
C	1.796188	0.1769962	1.7626075	C	-0.9578732	3.9692837	-1.4183165
C	1.3793972	0.6417389	3.0374421	H	-1.6348812	4.4119332	-2.1395245
C	2.2831789	1.3785469	3.8117116	C	-0.1064896	4.7707197	-0.6684669
H	1.9736077	1.7429236	4.7888628	C	0.7299307	4.0863471	0.2378285
C	3.5713678	1.6499645	3.3568856	H	1.4322514	4.6196653	0.8703711
H	4.2586282	2.223752	3.9737266	C	0.6815325	2.7109247	0.3498072
C	3.978238	1.1752178	2.1122937	H	1.3282505	2.1998069	1.0489445
H	4.9886582	1.3813159	1.7658853	C	-4.5753221	1.4847265	-4.7206903
C	3.1131478	0.433721	1.2992425	C	-5.969388	0.9907509	-4.2639871
C	-0.0208835	0.3637861	3.5700379	H	-6.7166942	1.2231535	-5.0313193
H	-0.5453688	-0.2202211	2.8080702	H	-6.2706494	1.4782286	-3.3306395
C	-0.8146763	1.6658502	3.797112	H	-5.9785861	-0.091392	-4.1001725
H	-0.334016	2.2983278	4.5531516	C	-4.198046	0.7824246	-6.0475209
H	-1.8267713	1.4339974	4.1487762	H	-4.943251	1.0147538	-6.8169349
H	-0.9008729	2.2478984	2.8739813	H	-4.1611753	-0.3051796	-5.9303697
C	0.0217921	-0.4705337	4.8652634	H	-3.2185562	1.1191602	-6.4035429
H	0.5417698	-1.419203	4.705533	C	-4.6520876	3.0003463	-4.974052
H	-0.9969721	-0.6921721	5.2041354	H	-3.6956132	3.4005908	-5.3291269
H	0.5328929	0.0703449	5.6709583	H	-4.9451792	3.5476729	-4.0709118
C	3.5949988	-0.0693994	-0.0559066	H	-5.4037111	3.2033568	-5.7442353
H	2.7648127	-0.6188264	-0.5093822	C	-0.0507554	6.2942899	-0.7887383
C	3.963193	1.0936137	-0.9987136	C	1.379156	6.7185514	-1.2027143
H	4.274814	0.7037127	-1.9747427	H	1.4380372	7.8107482	-1.2723327
H	4.7925468	1.6863165	-0.5943303	H	1.6458364	6.2962138	-2.1774062
H	3.1129617	1.7647821	-1.1568191	H	2.1239478	6.3856669	-0.4729845
C	4.7849561	-1.0379237	0.0901651	C	-0.3889518	6.9240075	0.5842727
H	5.0845987	-1.417379	-0.8937426	H	-0.3323192	8.0164819	0.5170229
H	4.521857	-1.8928022	0.7193626	H	0.3105991	6.5962676	1.3596708
H	5.6536706	-0.5378089	0.5353061	H	-1.4008165	6.6502864	0.9016779
C	-2.3963449	-0.5529427	-2.3197989	C	-1.0439464	6.8325285	-1.8332338
H	-2.2106562	-1.5754322	-2.0163865	H	-2.0792552	6.5841927	-1.573301
C	-3.3272221	-0.2208779	-3.2825136	H	-0.8320287	6.4392349	-2.8339893
H	-3.88973	-1.0242562	-3.7473844	H	-0.9661305	7.9238047	-1.8817169
C	-3.5407942	1.1271923	-3.6515605				
C	-2.764735	2.0707571	-2.9908534				

Table S4a. Orbital contributions and calculated energies for (ap^{Ph})Ni(bpy^{t}Bu$_2$) (**3**) as determined by TPSS/def2-TZVP DFT computations.

3 (ap^{Ph})Ni(bpy^{t}Bu$_2$)

	% Contribution			
**Orbital**	**Ni**	**Donor**	**Acceptor**	**Energy / eV**
LUMO+4	0	100	0	-1.219
LUMO+3	1.70	0.00	98.30	-1.696
LUMO+2	3.28	0.00	96.72	-1.969
LUMO+1	50.7	26.1	23.2	-2.494
LUMO	10.37	15.29	74.34	-2.777
HOMO	8.73	72.04	19.24	-3.476
HOMO-1	72.5	27.5	0.0	-4.49
HOMO-2	93.3	5.4	1.9	-4.703
HOMO-3	66.6	25.0	8.5	-4.955
HOMO-4	11.38	88.62	0.00	-5.056

Table S4b. Optimized coordinates of (ap^{Ph})Ni(bpy^{t}Bu$_2$) (**3**) in XYZ format.

Energy		-3455.247365	
Ni	-0.0456218	0.6249757	0.4466064
O	-0.7142483	1.9646761	1.4956617
N	-1.7719559	0.63641	-0.3397363
N	1.3792365	0.3785154	1.6714184
N	1.0705048	-0.4647416	-0.6859967
C	-1.999981	2.2352277	1.3050882
C	-2.6323029	1.5565854	0.2604738
C	-4.045372	1.7935784	0.0144185
C	-4.8096365	1.0982759	-0.9586244
C	-6.1583662	1.3384063	-1.1501268
C	-6.8201564	2.295028	-0.368357
C	-6.1099938	2.977818	0.6020754
C	-4.7346296	2.7582588	0.8335086
C	-4.0382972	3.4732316	1.8944589
C	-4.6591574	4.4433334	2.712529
C	-3.9715019	5.0931594	3.7242637
C	-2.6154879	4.8004715	3.9580802
C	-1.9727113	3.8644716	3.1720828
C	-2.6645827	3.1864378	2.1400832
C	-2.0730803	0.0312336	-1.5846229
C	-2.0253089	0.7892091	-2.7872237
C	-2.2607988	0.1318991	-4.0000366
C	-2.5403232	-1.2323099	-4.0449009
C	-2.5831388	-1.9660226	-2.8617014
C	-2.3478417	-1.361754	-1.6223115
C	-1.6997224	2.2794707	-2.7955823
C	-0.3364255	2.5531478	-3.4637733
C	-2.8063722	3.1062329	-3.4792921
C	-2.4348821	-2.1898552	-0.3460701
C	-1.5228912	-3.4302029	-0.388758
C	-3.8911577	-2.6058385	-0.0520981

C	1.4481343	0.9181917	2.9115434	H	-2.8946922	2.8536067	-4.5427258
C	2.4920622	0.6588823	3.7759786	H	-2.5710422	4.1743655	-3.4093333
C	3.5497613	-0.2001075	3.3988387	H	-2.0953685	-1.5479399	0.4727805
C	3.4733011	-0.7395243	2.1201602	H	-0.4796742	-3.153621	-0.5700514
C	2.3941242	-0.4451591	1.2738331	H	-1.8298824	-4.1296232	-1.1755719
C	2.2177199	-0.9330164	-0.0758261	H	-1.5739037	-3.965171	0.5667071
C	3.116546	-1.7708407	-0.7400613	H	-4.5438808	-1.7328832	0.0400865
C	2.9235843	-2.1463696	-2.0685716	H	-3.9404806	-3.1709002	0.8861803
C	1.7767053	-1.6187137	-2.6866991	H	-4.2864617	-3.244014	-0.8518723
C	0.8981603	-0.8128158	-1.9810089	H	0.6210565	1.5679563	3.1710404
C	4.6956169	-0.4929351	4.3700827	H	2.4748404	1.1270996	4.7550506
C	5.7415863	-1.4467955	3.7670929	H	4.2524718	-1.3986668	1.755568
C	4.1209601	-1.1413004	5.6528763	H	3.9872886	-2.1223185	-0.1954505
C	5.3981086	0.8347871	4.743709	H	1.5448313	-1.8278279	-3.7243944
C	3.919596	-3.0686289	-2.7779766	H	0.0128815	-0.4243209	-2.4661135
C	3.513175	-3.3416332	-4.237198	H	5.3032794	-2.4176702	3.5092402
C	3.9874136	-4.4192235	-2.0253473	H	6.5367739	-1.6250332	4.4986807
C	5.3214653	-2.4133598	-2.7725179	H	6.2054031	-1.0238317	2.8686501
H	-4.3333645	0.351935	-1.5776831	H	3.6255791	-2.0906677	5.4230687
H	-6.6988738	0.7772468	-1.908067	H	3.3918297	-0.487583	6.1418107
H	-7.8787048	2.4935886	-0.5122152	H	4.9301665	-1.3381978	6.3654652
H	-6.6377529	3.7015084	1.2140821	H	5.8251547	1.3143849	3.8565317
H	-5.7016988	4.6981724	2.5531709	H	6.2091831	0.6402865	5.4548167
H	-4.4825817	5.8337664	4.3335751	H	4.7027992	1.5402218	5.2095539
H	-2.0750524	5.315191	4.7484367	H	2.5351581	-3.8311721	-4.2999308
H	-0.921314	3.6407248	3.3216675	H	4.2496648	-4.0059647	-4.701772
H	-2.2250771	0.7000032	-4.9268459	H	3.477364	-2.419014	-4.8269835
H	-2.7263781	-1.7205329	-4.9982232	H	3.0094253	-4.9119035	-2.0170361
H	-2.8086843	-3.0292739	-2.9008268	H	4.3086939	-4.2848622	-0.9871885
H	-1.628713	2.6113553	-1.7565439	H	4.7051082	-5.0856333	-2.5176236
H	0.4761289	2.0380355	-2.9411598	H	5.3077658	-1.4553	-3.3028938
H	-0.1191854	3.6273684	-3.4487643	H	6.0432487	-3.0718637	-3.2693319
H	-0.3346666	2.2231846	-4.5095991	H	5.6769418	-2.2322327	-1.7528654
H	-3.7774214	2.9372541	-3.0055413				
